# Parent-mediated intervention versus no intervention for infants at high risk of autism: a parallel, single-blind, randomised trial

**DOI:** 10.1016/S2215-0366(14)00091-1

**Published:** 2015-01-22

**Authors:** Jonathan Green, Tony Charman, Andrew Pickles, Ming W Wan, Mayada Elsabbagh, Vicky Slonims, Carol Taylor, Janet McNally, Rhonda Booth, Teodora Gliga, Emily J H Jones, Clare Harrop, Rachael Bedford, Mark H Johnson, the BASIS team

**Affiliations:** a Institute of Brain, Behaviour and Mental Health, University of Manchester, Manchester, UK; b Institute of Psychiatry, Psychology and Neuroscience, King's College London, Department of Psychology, London, UK; c National Institute for Health Research Mental Health Biomedical Research Centre, South London and Maudsley NHS Foundation Trust, London, UK; d Centre for Brain and Cognitive Development, Birkbeck College, London, UK; e Department of Psychiatry, McGill University, West Montreal, QC, Canada; f Evelina London Children's Hospital and Kings College London Neurosciences Centre, London, UK; g Institute of Psychological Sciences, University of Leeds, Leeds, UK; h Institute of Child Health, University College London, London, UK; i Kasari Lab, UCLA Center for Autism Research and Treatment, Semel Institute, Los Angeles, CA, USA

## Abstract

**Background:**

Risk markers for later autism identified in the first year of life present plausible intervention targets during early development. We aimed to assess the effect of a parent-mediated intervention for infants at high risk of autism on these markers.

**Methods:**

We did a two-site, two-arm assessor-blinded randomised controlled trial of families with an infant at familial high risk of autism aged 7–10 months, testing the adapted Video Interaction to Promote Positive Parenting (iBASIS-VIPP) versus no intervention. Families were randomly assigned to intervention or no intervention groups using a permuted block approach stratified by centre. Assessors, but not families or therapists, were masked to group assignment. The primary outcome was infant attentiveness to parent. Regression analysis was done on an intention-to-treat basis. This trial is registered with ISCRTN Registry, number ISRCTN87373263.

**Findings:**

We randomly assigned 54 families between April 11, 2011, and Dec 4, 2012 (28 to intervention, 26 to no intervention). Although CIs sometimes include the null, point estimates suggest that the intervention increased the primary outcome of infant attentiveness to parent (effect size 0·29, 95% CI −0·26 to 0·86, thus including possibilities ranging from a small negative treatment effect to a strongly positive treatment effect). For secondary outcomes, the intervention reduced autism-risk behaviours (0·50, CI −0·15 to 1·08), increased parental non-directiveness (0·81, 0·28 to 1·52), improved attention disengagement (0·48, −0·01 to 1·02), and improved parent-rated infant adaptive function (χ^2^[2] 15·39, p=0·0005). There was a possibility of nil or negative effect in language and responsivity to vowel change (P1: ES–0·62, CI −2·42 to 0·31; P2: −0·29, −1·55 to 0·71).

**Interpretation:**

With the exception of the response to vowel change, our study showed positive estimates across a wide range of behavioural and brain function risk-markers and developmental outcomes that are consistent with a moderate intervention effect to reduce the risk for later autism. However, the estimates have wide CIs that include possible nil or small negative effects. The results are encouraging for development and prevention science, but need larger-scale replication to improve precision.

**Funding:**

Autistica, Waterloo Foundation, Autism Speaks, and the UK Medical Research Council.

## Introduction

Evidence from prospective studies suggests that about 20% of infants who have an older sibling with autism spectrum disorder (ASD) develop ASD themselves,^1^ and a further 20–30% develop broader social and communication-development disorders.^2^ Several specific infant behavioural and neural atypicalities have been identified during the first year of life associated with this later diagnosis of ASD; these include reduced behavioural attention to social scenes,^3^ declining attention to eyes,^4^ and attenuated neural response to eye gaze,^5^ and from 14 months altered attention disengagement^6^ and atypical infant temperament.^7^ These early developmental markers of later ASD are paralleled by reported perturbations in parent–infant interactions from at least 8 months of age in high-risk compared with low-risk parent–infant dyads.^8^ These perturbations are associated with infant atypicalities in infant gaze processing,^9^ and by age 14 months are themselves predictive of ASD diagnosis at 3 years.^10^ Taken together, these findings suggest that initial neurodevelopmental atypicalities in ASD, associated with changes to dyadic interaction with caregivers might represent increasingly atypical trajectories on the path to later ASD diagnosis. Such a model does not imply that interaction cycles are a cause of ASD, but that altered social interactions might maintain or perhaps amplify pre-existing vulnerability. This is consistent with findings from studies of neurotypical development on the importance of parent–child interaction quality for later socialisation and communication.^11^ In the context of atypical neurodevelopment, Down's syndrome, cerebral palsy, and learning disabilities can all be associated with altered parental responding and raised directiveness towards the child, plausibly because parents struggle to interpret accurately infants' behaviours or because the children have poorly regulated interaction and attention.^12^, ^13^ Relevant parental skills in social interaction with infants can be improved with intervention,^14^ and a related intervention was successful in doing this with children diagnosed with ASD between 2 years and 4 years 11 months.^15^ In the context of this evidence and theory, our study tested the effect of a very early intervention (at age 9–14 months), aiming to optimise social interaction for infants at high risk in the infancy prodrome of ASD. The logic of intervening this early within the first year derives from the research suggesting that atypical developmental trajectories towards ASD are emergent during this period, with potential developmental plasticity during this time of accelerated social and general learning in normal development.^16^ Intervention during this prodromal phase of ASD aims to change risk or severity trajectories before diagnosis. Experimental randomised trials of targeted interventions in such a context can also inform investigation of causal effect in developmental science; for instance, whether the interactional associations with atypicality are merely an epiphenomenon (with no bearing on future development), or whether they might have adverse consequences.^17^, ^18^ Therefore our aim was to test the effect of a parent-mediated intervention for infants at high risk of ASD in an experimental trial, and to use this intervention study to test hypotheses about the sensitivity to environmental change of selected risk markers for later ASD.

The hypotheses were that a developmentally targeted environmental change (a structured psychosocial intervention in infants aged between 9–14 months) will modify the following early risk markers for ASD in infancy: (1) markers of atypical interaction (including infant attention to parent as a primary outcome), (2) early ASD-related behavioural atypicality, and (3) neurophysiological biomarkers (attention disengagement and event-related potential to speech sounds).

## Methods

### Study design and participants

We decided to do a two-site (London and Manchester, UK)prevention randomised controlled trial of two parallel groups: intervention and no intervention. We screened siblings of autistic probands sampled within the context of the prospective longitudinal observational British Autism Study of Infant Siblings (BASIS), age 7–10 months at baseline. Exclusion criteria were any substantial medical disorder in the infant, being a twin, prematurity of less than 34 weeks, or a birthweight of less than 5 lbs (2·27 kg). The families were approached in order of identification and infants were not selected on the basis of developmental characteristics or atypicality. Families were paid travel expenses for research visits, but no other remuneration or incentive was given. Therapists were graduate speech and language therapists and psychologists, trained and supervised at two centres (Evelina Children's Hospital, London, and University of Manchester, Manchester). The study was approved by the London Research Ethics Committee (09/H0718/14, April 23, 2009); each family provided written informed consent.

### Randomisation and masking

Participants were enrolled by trial administrators at Birkbeck College and University of Manchester. After consent and baseline assessment, family details were registered at the Manchester trial office, and their identification number and centre telephoned to an independent statistician at the Christie Clinical Trials Unit in Manchester. We randomly assigned families (1:1) to either intervention or no intervention, stratified by centre (London or Manchester), using a permuted block approach within the two strata with random block sizes of four or six generated by the Clinical Trials Unit statistician. The statistician informed the trial office and clinical teams of allocation by telephone and email. Assessments were made at pre-randomisation baseline and after 5 months of treatment. Assessors and supervising research staff were independent from therapists, housed in different buildings, and were unaware of treatment allocation and the method of randomisation. Treatment allocation could not be masked from families and therapists. Assessors administered and coded all assessments with other information concealed, including group allocation, with the exception of parent-rated measures of language and adaptation.

### Procedures

Our intervention was a modification of the Video Interaction for Promoting Positive Parenting (VIPP) programme,^19^ which works with parents using video-feedback to help them to understand and adapt to their infant's individual communication style to promote the best possible social and communicative development. In the series of home-based sessions, the therapist makes videotapes of interactions between the parent and child in the home setting and uses video excerpts to work with the parent in a series of sessions that are developmentally sequenced to improve the quality of parent understanding of infant's communication. The focus is first on interpretation of the infant's behaviour and recognising their intentions, then working on sequences of sensitive responding during everyday activities, emotional attunement, and patterns of verbal and non-verbal interaction. The therapy has an evidence base for changing relevant aspects of parental interactive behaviour in infancy contexts other than autism.^19^ Because of the developmental complexity of prodromal ASD and the probable need for an increased intensity for ASD intervention, we extended the original VIPP programme from six sessions to a possible 12 by adding up to six planned booster sessions, according to need and in discussion with the family. We also developed additional therapeutic procedures to respond to any emerging developmental atypicality noted by the therapist (the trial protocol is available from the investigators and online). The theoretical rationale, feasibility, and acceptability of this extended manualised intervention (now called iBASIS-VIPP) and the therapeutic and assessment procedures used in this trial were demonstrated within a previous independent case series study.^20^ Two therapists (one at each site) did home interventions that were all videotaped. Therapist fidelity to the manual was assessed on 23 sessions from 15 participants, randomly selected to balance timepoint and therapist, and double-coded with a 21 pass or fail-item measure of therapeutic skills and specific iBASIS-VIPP strategies. Mean fidelity score was 19·4 passed items per session (93%, range 15–21), with only one of 23 sessions not meeting the pre-specified 80% fidelity threshold. The comparator group had no planned intervention. As part of their connection with BASIS, all families in both groups of the trial were involved in continuing lab visits and communication from the Birkbeck centre, but this did not constitute an intervention and was identical across all participants. All BASIS staff were masked to treatment allocation throughout the study.

Assessment data were obtained at the Centre for Brain and Cognitive Development, Birkbeck, London. We used the Manchester Assessment of Caregiver–Infant interaction (MACI),^9^ a validated global rating measure of 6 min videotaped free-play interaction between parent and infant, to measure infant attentiveness to parent and other interaction variables. The interactions took place in a research setting, with the parent asked to play with their infant as they usually do at home, with toys provided if needed. A trained reliable coder blind-rated the episode on two caregiver (sensitive responsiveness and caregiver non-directiveness), three infant (attentiveness to caregiver, infant affect, and infant liveliness), and two dyadic or interaction domains (mutuality and dyadic engagement intensity) along seven-point scales (see appendix and Green and colleagues^20^ for details). The MACI identifies interaction differences between at-risk and low-risk infants at 7 months,^8^ and low scores on three scales (infant attentiveness, positive affect, and dyadic mutuality) in high-risk infants at 14 months predict a diagnosis of ASD at age 3 years.^10^ Within-trial double coding of 38% of trial recordings showed good to high inter-rater agreement (single measures intraclass correlations by use of a two-way mixed effects model) ranging from *r*=0·64 to 0·75 (p<0·001).

We used the Autism Observation Scale for Infants (AOSI),^21^ a semistructured observational assessment of early behavioural risk markers for children with ASD, such as response to their name, social reciprocity, and imitation, and items assessing motor, attention and sensory behaviours, to measure infant atypical presymptom behaviour. AOSI total score at 14 months is associated with a diagnosis of ASD at 3 years.^7^

Attention disengagement was measured with the Gap-overlap task, using Tobii 1750/TX120 eyetrackers (Tobii Pro, Stockholm, Sweden). Matlab and the Talk2Tobii toolbox allowed for gaze-contingent stimulus presentation. When infants fixated on a central stimulus (a cartoon clock or balloon), a lateral target (cartoon cloud) was presented to the left or right at a visual angle of 15°. Saccadic reaction time was measured in baseline (where the central stimulus disappears at the onset of the lateral target) and overlap (where the central stimulus stays on screen during the presentation of the lateral target) trial types. A difference score (reaction time at overlap minus reaction time at baseline) was calculated for each infant and shows their ability to shift attention between visual stimuli under competition conditions (see appendix for further detail). An auditory oddball event-related-potential to speech sounds (ERP) paradigm was used, which measured the ability to detect and orient attention to changes in speech sounds. 77% of stimuli were /u/ vowels (standards), with two different types of infrequent (oddball) sounds, speech oddballs (/i/ vowels with the same pitch as the standards) and pitch oddballs (/u/ vowels with a different pitch to that of the standards), each presented with 11·5% probability. The infant was seated on caregiver's lap with visual distraction. Activity was measured with an EGI 128-channel Hydrocel Sensor Net (EGI, Oregon, USA). On the basis of results from Lepisto and colleagues,^22^ only the response to speech deviants was examined. Differences in response amplitude between oddballs and standards (the mismatch response) were calculated within two time windows showing discrimination (P1 around 150 ms) and attention orienting (P2 around 400 ms; see appendix for further detail). Mullen Scales of Early Learning (MSEL) is a standardised developmental assessment, which measures early motor, language, and cognitive development in children aged 0–68 months. Vineland Adaptive Behavior Scales (VABS-II) are parent-reported measures of adaptive behaviour yielding age-normalised competency levels on motor, communication, socialisation, and daily living skills domains. The MacArthur-Bates Communicative Development Inventory (MCDI) is a parent-reported measure of vocabulary and gestures.

### Outcomes

The primary outcome was infant attentiveness to parent. Secondary outcomes were atypical infant behaviour, parent-child interaction, direct and parent-reported language, event-related potentials to vowel change, attention disengagement, and adaptive function.

### Statistical analysis

We report full preplanned analyses for all participants. The target number of participants was 50, which would provide a study power of 80% (two-tailed α=0·05) to detect an outcome group difference of 0·8 SD for AOSI. Before outcome data inspection, analysis and treatment group unmasking, we revised the pretrial statistical analysis plan on the basis of new intervention data in older children with ASD diagnoses at 3 years^15^ showing good effect on child dyadic communication with parent but less on ASD symptom outcome, and new developmental data^10^ showing that the measure of infant dyadic attention to their parent at 14 months predicted ASD diagnosis later at age 3 years. We thus followed these data in changing the infant dyadic attention measure with parent (MACI) to our primary outcome and the measure of infant atypical presymptom behaviour (AOSI) to a secondary outcome.

Data preparation was undertaken with treatment assignment masked. Analysis was done with the program Stata version 13, which used uninformative treatment labels. Reported intention-to-treat (ITT) effect estimates correspond to group differences from regression analyses of the endpoint variable that covaried for the corresponding pre-randomisation baseline variable, age at endpoint assessment for all measures that were not standardised by age, and variables for which descriptive data suggested imbalance (>0·25 SD) at baseline. Quantile–quantile plots of residuals suggested skew-minimising transformations of the MCDI Receptive and Expressive vocabulary as necessary, for which additionally, because of expected floor effects, no covariation by baseline MCDI had been planned. Endpoint measures with several scales were analysed as a multivariate set by use of seemingly unrelated regressions,^23^ allowing regressions with different responses and covariates to be estimated together while accounting for their correlation (by use of the Stata sureg program). This reduced the difficulty of multiple-testing by allowing overall tests across several treatment effect estimates, exploited the correlation across measures for improved efficiency, and, estimated by maximum likelihood, made the missing-at-random assumption more plausible. We estimated effect sizes on the basis of a pooled estimate of the within group variance of each endpoint outcome, with percentile-based CIs estimated with bootstrapping (1000 replicates). Insufficient baseline data were missing to need imputation. Because data were missing for 35% of the participants in the ERP experiment, an inclusion rate similar to other ERP studies undertaken with 1 year old infants,^24^ for this analysis only we undertook an unplanned adjustment using propensity scores^25^ based on the two variables most significantly associated with treatment group in this subsample (maternal qualifications and baseline MCDI) to form participants into four strata that were balanced (Stata pscore). These strata were included as a four-level factor in the analysis.

### Role of the funding source

The funders of the study had no role in study design, data collection, data analysis, data interpretation, or writing of the report. The corresponding author had full access to all the data in the study and had final responsibility for the decision to submit for publication.

## Results

Between April 11, 2011, and Dec 4, 2012, we randomly assigned 54 families to treatment—28 to intervention, 26 to no intervention (the control group). The recruitment of 54 families exceeded our target of 50. Table 1 shows that the two treatment groups had similar characteristics at baseline, apart from maternal ethnic origin, maternal qualifications, and sex, which were included as analysis covariates.

**Figure 1:**
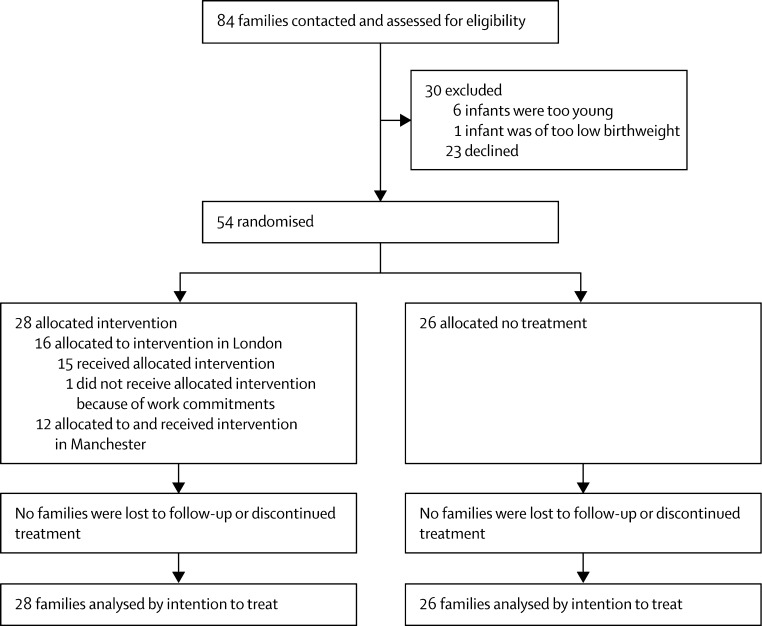
Trial profile

Figure 1 shows the flow of participants through the trial. 28 families were randomly assigned to receive the intervention, but one of these families (in London) could not begin treatment because of personal commitments. All 27 families who began treatment (12 in Manchester, 15 in London) completed the six core sessions over 5 months, with a mean of 9·5 of 12 possible sessions attended per family (SD 1·6, range 6–11). No adverse effects from intervention were reported and no site effects were found on analysis.

**Figure 2:**
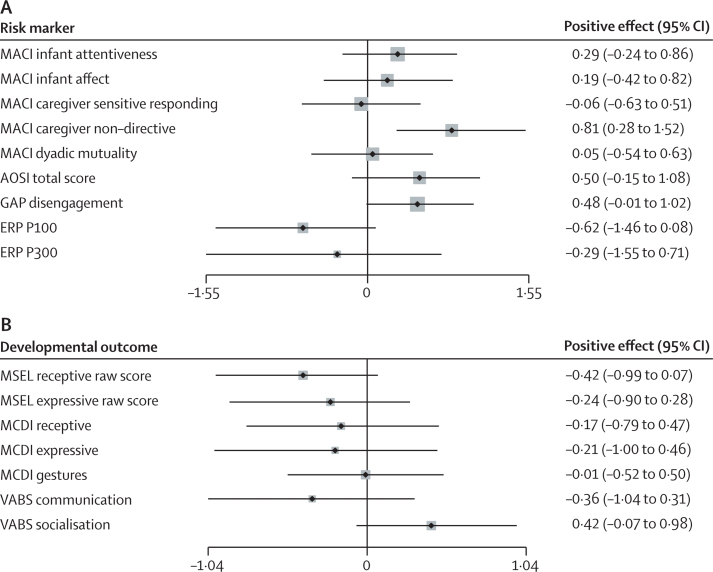
Effects on (A) postulated risk markers targeted for intervention change and (B) other measured developmental outcomes MACI=Manchester Assessment of Caregiver-Infant interaction. AOSI=Autism Observation Schedule for Infants. GAP disengagement=GAP-overlap attention disengagement task. ERP=event-related potential to speech sounds. MSEL=Mullen Scales of Early Learning. MCDI=MacArthur-Bates Communicative Development Inventory. VABS=Vineland Adaptive Behaviour Scales. Because lower scores represent improvement for AOSI and GAP, the effect sizes reported throughout are for reversed scales.

Table 2 shows baseline and end-of-trial scores for all primary and secondary outcomes and figure 2 a representation of treatment effect size point estimates and associated CIs.

For the primary outcome, infant attentiveness to parent, regression analysis adjusted for baseline variables that were not balanced between groups at baseline showed a slight ITT estimate of β 0·31 (CI −0·30 to 0·93), with point estimate effect size of 0·29 with 95% CI) of −0·24 to 0·86, thus including possibilities ranging from a small negative treatment effect to a strongly positive treatment effect. A group difference in slope suggesting greater efficacy for higher levels of baseline infant attentiveness was not significant (p=0·16).

The four secondary interaction outcomes gave strong evidence for a significant intervention effect (χ^2^(4)=13·44, p=0·009), largely due to increased caregiver nondirectiveness (0·81, 95% 0·28, 1·52). Improvements were also seen in behavioural atypicality on the AOSI total score, which improved by 2·51 points (effect size 0·50, 95% CI −0·15 to 1·08) and faster disengagement in the Gap-overlap task (effect size 0·48, 95% CI −0·01 to 1·02). The overall significant effect on parent-reported adaptive behaviour (χ^2^(2)=15·39, p=0·0005) arose from improved social adaptation (effect size 0·42, 95% CI −0·07 to 0·98) but perhaps some reduced communication (effect size −0·36, 95% CI −1·04 to 0·31). No overall intervention effects were found for auditory ERPs (χ^2^(2)=2·23, p=0·33), directly assessed Mullen language scales (χ^2^(2)=2·42, p=0·30) or parent-reported MCDI vocabulary scores (χ^2^(2)=0·57, p=0·75).

## Discussion

This is the first report of a randomised early intervention trial in the first year of life for infants at high risk of ASD (panel). We used prespecified analyses to test the effect of a targeted, parent-mediated, social-communication intervention on established risk markers in infancy for later ASD diagnosis. Infants were not selected for atypicality. The trial shows the feasibility of delivering and testing an early prodromal intervention of this kind, because all families who started the intervention completed it successfully. The effect sizes shown in our trial are moderate by the scale of psychosocial trials and generally consistent in direction across our different domains of measurement (from behaviour and social interaction to brain function), with exceptions in language and auditory ERP. The precision of these estimates is limited by our sample size; in most cases the 95% CIs around the point estimates include a small negative effect of treatment and positive effects. These results therefore, although exciting in the context of intervention science for ASD, need replication on a larger sample before making more precise or definite conclusions.

There was a slight point estimate intervention effect to improve infant attentiveness to caregiver, with 95% CI including estimates ranging from a slight negative effect to a large positive effect. Independent prospective study has shown that reduced infant attentiveness on this measure at 14 months is associated with later diagnosis of ASD,^10^ and this is similar to other reports on early infant social attention.^3^, ^4^ To the extent that attentiveness is improved at the 14 month endpoint, therefore the intervention is plausibly acting to ameliorate an established early marker of ASD emergence.

The intervention showed a strong effect to increase caregiver non-directiveness. Previous studies have shown high parental directiveness in high-risk infants compared with low-risk infants;^8^, ^9^ as noted in the Introduction, this occurs with other disabilities and might be due to parents struggling to interpret accurately infants' behaviours or children's poorly regulated interaction and attention.^12^, ^13^ From developmental theory, such directiveness could be associated with adverse developmental outcomes for the infant, and be part of an amplifying atypical interactional trajectory in prodromal ASD. For this reason, the parent-mediated intervention targeted a modification of such interactional styles and the trial data show that it has succeeded. By contrast, the intervention did not show the postulated effect on the (also targeted) caregiver sensitive-responding or dyadic mutuality. A trial of differently modified VIPP intervention in older children with diagnosed ASD^27^ reported a similar pattern of effect on parent interaction behaviours (although no effect on the outcome of child responsiveness to parent).

The point estimate effect on reduction of ASD-related atypical behaviours on AOSI (0·5), which has CIs ranging from a large reduction to a small increase, is potentially important because this measure is designed to identify the earliest manifestations of ASD symptoms,^21^ and high AOSI scores at 14 months are known to predict 3 year ASD outcome.^7^ The 4·15 point mean reduction found within the intervention group, compared with 1·77 in controls (2·38 point difference) is substantial in the context of the range of AOSI scores at 14 months. For example, an independent study showed a 1·62 point difference in mean AOSI scores at 14 months between high-risk infants later developing ASD diagnosis at 3 years and those not developing diagnosis.^10^ The size of reduction here contrasts with a 2-point reduction found within a recent case series (n=7) at a similar infant age.^26^ The data therefore suggest the possibility that this intervention might be able to modify in the short term the emergence of atypical ASD-related behaviours during development. This finding has added potential because later interventions after diagnosis have not yet been able to effect meaningful change in ASD symptoms;^10^, ^27^ and early intervention in infancy could benefit from the greater developmental plasticity at this age.^16^

The moderate-sized positive point estimate of effect on the GAP-overlap attention disengagement task includes CIs ranging from a very small negative effect to a large positive effect. The finding is novel, with important potential implications, suggesting that the change in caregiver–infant social interaction at this age through intervention has resulted in raised infant attentional flexibility or processing speed in association with (non-social) stimuli. The mean decrease in disengagement time in the treatment group is 50 ms; a large change in the context of developmental studies which show that a plateauing or a progressive increase in disengagement time over this period in infancy is the most replicated early marker for later ASD diagnosis up to now.^6^, ^28^ Attentional flexibility of this kind has been shown to have cross-domain effects on social competency;^29^ if substantiated, this effect would therefore be again in the direction of plausibly ameliorating a developmental trajectory towards ASD.

By contrast, the intervention shows either no effect or tendency towards slower progress on developmental language measures. This outcome is consistent with the findings on auditory ERP, which (although complicated by missing data and adjustment for group imbalance) show effect estimates suggesting reduced responsiveness to language sounds in the intervention group. The confidence intervals here include very large negative effects and very small to substantial positive effects. These unexpected findings were not driven by idiosyncratic case-level effects in the data. If they are not due to chance and are supported by later developmental follow-up, a number of possible explanations need consideration. Direct adverse effect of the intervention on language, mediated for instance by the rise in parental non-directiveness, is possible, but there is no association in the data between the increase in non-directiveness and language outcome and such a finding would be inconsistent with previous developmental or intervention literature about neurotypical infants.^11^, ^30^ The findings could alternatively suggest atypical pathways for language learning in these at-risk infants. They could alternatively show the effect of acceleration in one domain of development temporally slowing progress in another, or possibly relate to the baseline imbalance in ethnicity. These and other possibilities would need further careful consideration in the context of longer-term follow-up.

Overall, there is a notable pattern in the results here compared with similar interventions in later development. Whereas other parent-mediated and early social communication interventions in children under 5 years of age diagnosed with ASD tend to show greatest effects on target outcomes proximal to treatment, such as parental or child dyadic behaviours, with diminishing effects on more distal ASD symptoms,^15^ here signals of intervention effects are spread generally across parental, infant dyadic, symptom, and cognition outcomes. This spread possibly suggests a more generalised pattern of effect from intervention into cognition and brain function at the infancy stage, in accordance with theory related to early plasticity.^16^

Several factors could affect the generalisability of these results. In common with other studies with infants at high risk of ASD, the sample contained self-referrals and clinic referrals, and consequent selection biases cannot be excluded. The whole sample showed high levels of income and maternal qualification and replication studies would benefit from wider socioeconomic sampling. Again intrinsic to all high-risk infancy studies, the families had an older sibling with ASD, and generalisation of our results to infants developing ASD without this family history cannot be assumed. Relevant factors here could theoretically be the parents' experience of having had a typically developing infant and possible differences in ASD development within simplex and multiplex families. Prodromal interventions on population-based cohorts have not so far been feasible because of the absence of sensitive or specific screening methods for risk in infancy.

For the **trial protocol** see http://www.bbmh.manchester.ac.uk/ibasis

## Figures and Tables

**Table 1:** Baseline data

		**No intervention (control) (n=26)**	Intervention (n=28)*
Maternal medical history			
	No disorder	15 (58%)	17 (61%)
	Mental health or physical disorder	11 (42%)	11 (39%)
Maternal ethnic origin			
	White	22 (85%)	18 (64%)
	Other	4 (15%)	10 (36%)
Maternal qualifications			
	≥degree	15 (58%)	10 (37%)
	<degree	11 (42%)	17 (63%)
Annual household income			
	<£40 000	15 (58%)	16 (59%)
	≥£40 000	11 (42%)	11 (41%)
Sex			
	Male	12 (46%)	17 (61%)
	Female	14 (54%)	11 (39%)
Typical older sibling(s)			
	TD sib(s)	15 (58%)	14 (50%)
	No TD sib(s)	11 (42%)	14 (50%)
Age (days)		276·58 (24·25)	267·14 (20·93)
MSEL nonverbal T-score		57·29 (10·69)	53·71 (12·73)

Data are n (unimputed sample %) or mean (SD) for available cases. TD=typically developing. MSEL=Mullen Scales of Early Learning.

* n=27 for maternal qualifications and annual household income.

**Table 2:** Baseline and endpoint data by treatment group

	**No intervention**			**Intervention**		
	Baseline	Endpoint	Change	Baseline	Endpoint	Change
MACI infant attentiveness	3·65 (1·29), n=26	4·19 (1·13), n=26	0·54 (1·77), n=26	3·39 (1·26), n=28	4·22 (1·05), n=27	0·81 (1·49), n=27
MACI infant affect	4·08 (1·13), n=26	4·84 (1·38), n=26	0·77 (1·73), n=26	4·18 (1·19), n=28	4·85 (1·23), n=27	0·67 (1·57), n=27
MACI caregiver sensitive responding	3·85 (1·19), n=26	4·58 (1·10), n=26	0·73 (1·46), n=26	3·68 (0·98), n=28	4·30 (1·20), n=27	0·59 (1·25), n=27
MACI caregiver non-directiveness	3·73 (1·43), n=26	3·92 (1·32), n=26	0·19 (1·90), n=26	3·50 (1·48), n=28	4·67 (1·24), n=27	1·19 (1·78), n=27
MACI dyadic mutuality	3·00 (1·06), n=26	3·46 (1·30), n=26	0·46 (1·63), n=26	2·86 (1·21), n=28	3·22 (1·05), n=27	0·37 (1·42), n=27
AOSI total score	9·08 (5·32), n=26	7·31 (5·83), n=26	−1·77 (6·98), n=26	10·04 (4·60), n=28	5·93 (4·05), n=27	−4·15 (4·67), n=27
MSEL receptive raw score	10·81 (1·86), n=26	15·46 (3·25), n=26	4·65 (3·75), n=26	10·43 (1·67), n=28	13·81 (1·75), n=27	3·37 (2·59), n=27
MSEL expressive raw score	10·73 (1·73), n=26	15·42 (2·93), n=26	4·69 (3·30), n=26	10·21 (2·17), n=28	14·41 (2·00), n=27	4·15 (2·46), n27
MCDI receptive	67% non-zero=24	121·8 (71·02), n=25	..	44% non-zero=27	92·54 (83·69), n=26	..
MCDI expressive	32% non-zero=25	24·6 (43·13), n=25	..	22% non-zero=27	14·77 (22·90), n=26	..
MCDI gestures	7·33 (4·99), n=24	31·04 (13·75), n=25	22·5 (10·18), n=24	6·07 (4·80), n=27	29·44 (12·80), n=25	21·96 (10·34), n=24
VABS communication	28·20 (4·47), n=25	30·04 (5·04), n=25	1·42 (4·85), n=24	27·93 (5·23), n=27	28·27 (3·74), n=26	−0·36 (4·68), n=25
VABS socialisation	30·04 (3·38), n=25	43·8 (6·48), n=25	13·17 (4·91), n=24	28·59 (5·02), n=27	44·58 (5·90), n=26	15·08 (4·80), n=25
GAP disengagement	182·79 (81·59), n=25	171·62 (100·39), n=24	−9·01 (83·44), n=23	209·74 (76·49), n=28	160·84 (81·75), n=26	−50·97 (78·95), n=26
ERP P100	..	1·27 (1·83), n=15	..	..	0·31 (1·71), n=20	..
ERP P300	..	2·57 (3·03), n=15	..	..	1·33 (3·43), n=20	..

Unimputed sample data are means (SD) for available cases. Scores at baseline for the MCDI showed such severe floor effects that we report the binary (zero, >zero). At endpoint, the most incomplete variables were missing 4 of 54 values. One case with unusually low VABS communication at baseline did not complete this measure at endpoint. ERP scores were available for only 15 (58%) of 26 from non-intervention and from 20 (71%) of 28 intervention participants, due to non-compliance at wearing the sensor net (n=7), insufficient artefact-free data (n=7), or technical errors (n=5). MACI=Manchester Assessment of Caregiver-Infant interaction. AOSI=Autism Observation Schedule for Infants. MSEL=Mullen Scales of Early Learning. MCDI=MacArthur-Bates Communicative Development Inventory. VABS=Vineland Adaptive Behaviour Scales. GAP disengagement=GAP-overlap attention disengagement task. ERP=event-related-potential to speech sounds.

## Panel Research in context

**Systematic review**

We found a systematic review published in 2014^31^ that identified only three small studies^20^, ^32^, ^33^ of intervention for infants at risk for autism starting in their first year of life. One was a case series with low-risk control (n=7)^20^ and two had multiple baseline designs (n=3).^32^, ^33^ Subsequently, a further small case series (n=7)^26^ has been published. There are thus no previous randomised controlled trials in the scientific literature of infants at high risk of developing autism. A prized goal of neurodevelopmental research has been for targeted environmental intervention to achieve integrated treatment effects across domains of behaviour, interaction, and developmental neuroscience. Our intervention study was built on research identifying characteristics of the very early prodromal period in ASD. It aimed to improve a number of developmental risk markers that have been shown to be atypical in first year infants who go on to develop ASD. The social communication intervention proved feasible and acceptable to deliver in the first year of life.

**Interpretation**

This trial adds to the evidence base as the first randomised controlled trial of an intervention beginning under 1 year in infants at high risk of developing autism. The findings show broadly positive effect estimates across a range of measures, and are consistent with an intervention with moderate effects on several ASD risk markers. This is an exciting conclusion in the context of ameliorating later ASD risk but one that is very far from being definitive, in view of the low precision of the estimates that the sample size allows. The trial is large by the standards of experimental infancy studies so far, but it is small by the standards of trials testing interventions that are practical in health services. Our results therefore need replication on a larger sample before more precise conclusions can be drawn or wider service inferences made.
