# Supplementary material for: Parent-mediated intervention versus no intervention for infants at high risk of autism: a parallel, single-blind, randomised trial
Source: Lancet Psychiatry. 2015 Jan 22;2(2):133–40. doi: 10.1016/S2215-0366(14)00091-1 (PMC4722333; doi:10.1016/S2215-0366(14)00091-1)
Supplement: Supplementary appendix [file mmc1.pdf]

THE LANCET Psychiatry

Supplementary appendix

This appendix formed part of the original submission and has been peer reviewed. We post it as supplied by the authors.

Supplement to: Green J, Charman, T Pickles A, et al. Parent-mediated intervention versus no intervention for infants at high risk of autism: a parallel, single-blind, randomised trial. *Lancet Psychiatry* 2015; published online Jan 22. http://dx.doi.org/10.1016/S2215-0366(14)00091-1.

PARENT-MEDIATED INTERVENTION FOR INFANTS AT HIGH RISK OF AUTISM: RANDOMISED CLINICAL TRIAL

Green J., Charman T., Pickles A., Wan MW., Elsabbagh M., Slonims V., Taylor C., McNally J., Booth R., Gilga T., Jones E., Harrop C., Bedford R., Johnson M.H.

ONLINE SUPPLEMENT

Table 1 - Manchester Assessment of Caregiver-Infant Interaction (MACI) scales

Methods 1 - ERP: Mismatch Response paradigm

Methods 2 – Gap-overlap task

TABLE 1 - MANCHESTER ASSESSMENT OF CAREGIVER-INFANT INTERACTION (MACI) SCALES

Domain *(Labels of extremes of the 1-7 scale)*	Brief definition
Caregiver	
Sensitive responsiveness *(minimally sensitively responsive to highly sensitively responsive)*	Contingent and appropriate behavioural responsiveness to infant behaviours to meet the infant's immediate, interactive and developmental needs, including an attentive attitude, warmth, and appropriate engagement, support and structuring in response to infant behaviour and/or lack of behaviour.
Nondirectiveness *(highly directive to highly nondirective)*	A behavioural and mental acceptance of and focus on the infant's experience, which promotes infant initiation, as opposed to demanding, intrusive, and negative behaviours and comments, which are directed by the caregiver's own agenda.
Infant	
Attentiveness to caregiver *(inattentive to highly attentive)*	Interest in the caregiver (as opposed to focus on other stimuli or self-absorption), through direct eye contact, acceptance of and interest in caregiver or joint activity, face/body orientation, and other references to caregiver activity, such as imitation.
Positive affect *(General/high negative affect to general/high positive affect)*	Displayed positive expression, vocalisation and behaviour, weighed against displayed negative affect as demonstrated by negative expression, vocalisation and bodily gestures.
Liveliness *(unlively to extremely lively)*	Amount and level of physical activity, particularly those behaviours initiated by the infant spontaneously, excluding reflex responses and movement controlled by the parent.
Dyadic	
Mutuality *(very low mutuality to consistently high mutuality)*	The amount and degree of dyadic reciprocity, attunement and 'togetherness', including shared attention, infant acceptance of caregiver involvement, playing together, interactive flow, and shared body orientation.
Intensity of engagement *(almost no engagement to very intense engagement)*	The intensity (not quantity) of engagement by both parties at its optimal point, directly or through mutual object focus, with higher intensity involving greater interest, arousal and/or positivity or excitement.

Within-trial double coding of 38% of trial recordings showed good to high inter-rater agreement (single measures intraclass correlations using a two-way mixed effects model) ranging from r = .64 to .75 ($p < 0.001$).

METHODS 1 - ERP: MISMATCH RESPONSE PARADIGM

Stimuli. The auditory stimuli were provided by Paul Iverson, University College London. They were processed versions of natural recordings of /i/ and /u/, produced by a male speaker. An overlap-add method within Praat[1] was used to equate the duration and the pitch contours of the syllables, and the amplitude envelope of the syllables were also equated. High- and low-pitched versions of these stimuli were then created by raising or lowering the pitch contour. The "low" pitch /u/ used as standard and /i/ used as vowel deviant had a natural falling pitch contour from 127 to 117 Hz. The "high" /u/, used as pitch deviant, was 10% higher.

Procedure. Infants were seated on their parent's lap facing and experimenter who blew soap bubbles throughout the study.

Recording and analysis. EEG was recorded using a 128-electrode Hydrocel Geodesic Sensor Net (EGI, Eugene, OR) with respect to the vertex electrode and sampled at 500 Hz. A 0.1-40Hz band-pass filter was applied offline before segmenting the recording into 800 ms long segments (100 ms pre-stimulus). Bad channels were marked by visual inspection and artefacted segments (> 20 bad channels) were rejected. Remaining bad channels were interpolated. Only participants with at least 10 trials in each condition were included in the analysis. There was no group difference in the participant exclusion criterions. Participants included in the analysis contributed in average 110 trials and there was no significant difference in the number of trials contributed by the two groups. Data was baseline corrected with respect to the 100ms pre-stimulus interval and re-referenced to the average reference. Electrodes and time windows were chosen for analysis based on visual inspection of the main differences between conditions in the whole group of participants. For the first time window (P1), voltage was averaged over the following sixteen electrodes: 112, 106, 105, 111, 13, 7, 30, 29, 36, 104, 42, 37, 31, 80, 87, 93, 6 and over the 220-340 ms. interval. For the second time window (P2), voltage was averaged over the following seventeen electrodes: 112, 106, 105, 111, 13, 7, 30, 29, 6, 20, 12, 5, 118, 11, 24, 36, 104, 124 and over the 350-550 ms. interval. The time windows and electrode locations are similar to those previously used with this paradigm and this age group.[2,3]

METHODS 2 – GAP-OVERLAP TASK

Procedure. Stimuli were presented on a Tobii 1750/TX120 eyetracker subtending 24˚; stimuli were presented gaze-contingently using Matlab and the Talk2Tobii toolbox. Stimuli were presented in five blocks interspersed with other elements of the testing battery. The first four blocks lasted 12 trials per block, with an 8-second video reward presented between trials 6 and 7; the fifth block continued until 12 usable trials per condition had been presented, until the infant became fussy or until 80 trials had elapsed. After fixating a Central Stimulus (a cartoon clock/balloon, subtending 4.5˚) a Lateral Target (a cartoon cloud, subtending 3˚) was presented to the left or right at an eccentricity of 6˚ following a delay of 150ms; when the participant looked to the LT they received a brief audio-visual reward. Reaction times were assessed in three conditions: (1) Gap: LT presented 200ms after the offset of the CS; (2) Baseline: CT offset simultaneous with LT onset; (3) Overlap: CS remained on screen when LT was presented. The start of each trial and the reward was automatically triggered online when gaze landed in the relevant area of the screen, following custom routines implemented in Matlab/Psychtoolbox. Reaction time was calculated as the time elapsed between LT appearance and the reported position of gaze entering the LT position (a 9˚ box around the LT). Trials were excluded from analysis if there was a period of more than 60ms of continuous data loss between peripheral stimulus onset and the eyes entering the position of the lateral target; if the eyes were not fixating the central stimulus at the time of peripheral stimulus onset; if the child did not make a saccade to the lateral target within 2 seconds of peripheral stimulus onset; or if the child disengaged from the screen within 2 seconds of peripheral stimulus onset without first saccading to the peripheral stimulus.

Subsequently, mean reaction time per condition was calculated, excluding reaction times less than 100ms (thought to be less than the minimum latency required to program a saccade in response a stimulus appearing) and reaction times greater than 1200 ms as they are thought not to represent exogenously driven reactions to the stimulus presentation.[4]

1. Boersma, P., Weenink, D. (2014). Praat: doing phonetics by computer [Computer program]. Version 5.3.80, retrieved 29 June 2014 from http://www.praat.org/
2. Dehaene-Lambertz, G., & Gliga, T. (2004). Common neural basis for phoneme processing in infants and adults. *Journal of Cognitive Neuroscience*,*16*(8), 1375-1387.
3. Kushnerenko, E., Ceponiene, R., Balan, P., Fellman, V., & Näätänen, R. (2002). Maturation of the auditory change detection response in infants: a longitudinal ERP study..*Neuroreport*, *13*(15), 1843-1848.
4. Elsabbagh M, Fernandes J, Webb S, Dawson G, Charman T, Johnson MH. Disengagement of visual attention in infancy is associated with emerging autism in toddlerhood. *Biol Psychiat.* 2013;74(3):189-194.

TRIAL PROTOCOL – JULY 15TH 2011

HYPOTHESES AND SPECIFIC AIMS

Aims

A pilot study to test the impact of a parent-mediated intervention for infants at high risk of autism. The study will gather information on:

a. Feasibility of recuitment and retention of subjects,
b. Feasibility of delivery of the intervention
c. Acceptability of the intervention for subjects and their adherence to the protocol;
d. Initial evidence of effect

Hypothesis

Can a developmentally targeted, environmental change (a structured psychosocial intervention) modify early behavioural markers of atypicality and targeted bio-markers related to brain function in a population of infants at-risk of autism spectrum disorder (ASD)?

BACKGROUND AND SIGNIFICANCE

Theoretical rationale

The “interactive specialisation” (IS) model[1] within cognitive neuroscience holds that that the “social brain” develops through an active process of postnatal interaction with the environment, (rather than through the innate linear unfolding of potential as proposed by a contrasting “maturational” hypothesis). The IS framework has successfully accounted for emerging evidence from studies of early face processing, oculomotor and attentional control, object processing, language, reading and developmental disorders.[2] IS postulates gradual changes in specialisation (the degree to which the responses of a cortical region are tuned to a certain class of input) and in localisation (the spatial extent of cortex activated following a stimulus presentation) in response to post natal activity. It predicts that:

1. Typically developing infants and toddlers are biased to orientate towards, attend to and learn from social stimuli. These biases contribute to the progressive post natal emergence of the typical cortical social brain network.
2. In autism spectrum disorders there is a failure or abnormality in one or more of the underlying mechanisms of biasing from early in life, which then disrupts the typical emergence of the social brain network. This trajectory becomes further compounded by atypical interactions with the environment leading to the established pattern of symptoms observable by the age of diagnosis, and later social cognitive and communicative functioning.

This IS model is complimentary with evidence within developmental psychology and psychopathology on the importance of the early social environment (particularly the estimated 1000 hours of one to one social interaction in the first year with parents/caregivers) on later social and communicative functioning: and how these early environments can be disrupted in

atypical development. In neurotypical infants, the quality of parent child interaction has been shown to have importance for later non-social contingency tasks[3] and the development of joint attention, reciprocity, mutuality, for later socialisation and communication[4-6]. Evidence suggests that atypical neurodevelopment (for instance in Down syndrome, cerebral palsy, learning disability) is often associated with generally reduced parental sensitivity and increased intrusiveness[7, 8]; this may arise from difficulties in accurately interpreting infants' behaviours.[9-11] While a more directive parental style is not incompatible with sensitivity[12], supportive, contingent and sensitive parental responses appear to be central to the development of joint attention; and later language development is accelerated when the adult follows child-initiated and child-focussed topics for joint attention.[12, 13] This may be particularly important in children whose developmental impairments lead to difficulties in accommodating demands to shift focus and to regulate several competing demands on attention.[14-16] The relevant parental skills can be trained.[17]

With regard to infants at high risk of autism, two important possibilities arise from these convergent theories:

1. If we can identify the precise character of the interactional 'perturbations' related to early developmental atypicality in autism, this may lead to the elucidation of adverse interactional cycles with a negative effect on later development. (This would be an example of an 'evoked' environmental response within a heritable disorder).
2. Intervention to improve these interactional cycles could in theory therefore positively impact on later developmental trajectories. (This would not suggest that these cycles were a primary cause of autism, but that they might maintain or amplify a pre-existing vulnerability).

Our work and others' suggests that systematic experimental trials of an intervention in this context can serve an additional function of being a powerful method for illuminating basic developmental theory.[18] It has historically been difficult within developmental research to identify directions of causality within reciprocal interactions; in the model above, for instance, it is not necessarily easy to know which interactional consequences of atypicality are simply an epiphenomenon (with no bearing on future development) or which could themselves act back on the developing child with adverse consequences on development. An experimental randomised trial of intervention combined with a repeated measures observational design creates two parallel groups of infants for follow up, one of whom has had an targeted intervention changing a proposed developmental mediator. This is a powerful method for investigating causal processes in development.[19]

Significance

As we outline below, our view is that both the basic neuroscience and the developmental psychopathology of autism are now at a stage where it is feasible and timely to initiate an integrated intervention/basic science study of this kind. Our proposed study represents a first step in this strategic line of research; which should allow the testing of competing predictions from the "interactive specialisation" and "maturational" hypotheses in specific aspects of autism development. The current study will provide tests of proof of concept and feasibility of the intervention and developmental measurement; plus initial systematic data to inform future larger scale studies in this area. It addresses the key issues relating to the early pathogenesis of autism and possibilities for strategic early prodromal intervention in the disorder.

PRELIMINARY DATA AND RATIONALE FOR THE STUDY

To rationally design and justify such an intervention study, we need to:

1. <u>Identify candidate areas of early atypicality indexing developmental risk which should be targets for change during intervention</u>. It is key to our design that these identified

targets for intervention are simultaneously developmental processes or biomarkers that hold promise as being implicated in the developmental pathogenesis of the disorder.

2. Identify known interactional perturbations associated with early atypicality. These will then be the focus for intervention.
3. Identify best practice intervention strategies to impact positively on these interactions. Intervention in this context must be theory and evidence based and sufficiently intensive and targeted to produce the necessary environmental change.

We set out here the preliminary data and rationale in relation to each of these areas.

1. Candidate areas of atypicality: behaviour and neurophysiology

Our candidate areas for this study include 1) patterns of behavioural atypicality that may provide a prodrome for the behavioural phenotype of the broader autism phenotype; 2) relevant and evidence based ‘biomarkers’ which may be associated with the pathogenesis of autism. (We define these biological markers, or biomarkers, as observable properties of an organism that indicate variation in cellular or biochemical components, structure, or function and that can be measured in biologic systems or samples). Our review[20] highlights evidence for a subtle and variable expression of behavioural atypicality in the first year; and contrasts this with somewhat more consistent results when more fine grain measures are used[21], and our results below). In this study we therefore investigate overt behavioural atypicality; but also combine this with measurement of more subtle expression that is detected using fine-grained behavioural measures and biomarkers.

a) Behavioural atypicality

Increasingly from the latter part of the first year and into the second, identified atypical behaviours in high risk autism sibs - in particular delayed emergence of early social communication behaviour such as joint attention, gaze monitoring and pretend play as well as abnormalities in attention - show association with later emergence of autism and the broader autism phenotype (BAP).[22-24] By BAP we include aspects of the behavioural phenotype (and in adults also including face processing[25], theory of mind[26], executive function[27, 28], and central coherence[29]) associated with the autism phenotype and found not only in affected individuals, but also (to a lesser degree) in their genetic relatives. BAP is increasingly reported in infant siblings in children with autism who do not go on to a full diagnosis.[20] Evidence of these behavioural markers of atypicality can now be found in systematic measurements as young as six months of age and thus provide a one index of developmental risk and change in the population of infants at high risk. Our study addresses these behaviours in the latter part of the first year: this timing balances the reduced predictive value at < 1 year with the advantage of earlier intervention in relation to developmental plasticity.

b) Neurophysiological and saccadic movement biomarkers

Neurophysiological biomarkers are important because they may reveal subtle patterns of atypical brain function not evident in overt behaviour until later in development. Further, from studies of typical development it is evident that neurophysiological analyses such as ERP and EEG can reveal stimulus or task-dependent effects of brain processing in pre-verbal infants that are not evident using standard behavioural measures; and recent developments in eye tracking technology allow the detection of subtle differences in processing not revealed with other behavioural assessments. For this study we have selected neurophysiological, attention and eye tracking biomarkers based on (1) addressing domains of cognition and brain function relevant to autism, (2) compatibility with an existing large-scale study of infants at-risk for autism (our UK Infant Sibs Phase 1 study already undertaken) to enable greater power for some comparisons, and (3) demonstrated sensitivity (or likely sensitivity) to environmental manipulation. We will use these biomarkers to assess specific hypotheses about the early developmental origins of autism, and its potential modulation by environmental manipulation.

We hypothesised earlier that in ASD there is a failure or atypicality in one or more of the underlying mechanisms of biasing toward social stimuli from early in life, which then disrupts the typical emergence of the social brain network. This trajectory becomes further compounded by atypical interactions with the environment leading to the established pattern of symptoms observable by the age of diagnosis. Based on the IS view, we further hypothesise that our intervention will prevent the compounding of early symptoms, without necessarily effecting the original atypical biasing to social stimuli in infants who go on to develop ASD. These hypotheses can be tested by assessing biomarkers of early social and non-social orienting and attention, and neurophysiological markers of cortical social stimulus processing. The modulation we predict is that only infants siblings assigned to intervention will go from atypical responses to stimuli at baseline to more age-typical responses at endpoint. Theory would suggest that there may be differential responses of these biomarker phenomena to environment intervention. Specifically it may be that the cortical social stimulus processing will be most responsive and non-social attentional control will be least sensitive to an intervention of the kind we will undertake. Identifications of such differential effects may be important for developmental theory and will also guide the more specific modeling of different intervention strategies to different deficits. There is already evidence that non-social attention and ERP measures of social processing can be influenced by experience in pre-school children. For example, Rueda et al[30] trained typically developing pre-school children in attention tasks and found later measurable changes in other attention measures and their ERP correlates, and de Haan et al[31] reported modulation of ERP face expression processing in infants reared by low affect mothers as compared to those reared by those with high affect scores.

Social attention and orienting. We will use a free-viewing task in which we simultaneously display five or six multiple objects including one face.[32] An eye tracking system registers the shifts in gaze of infants in response to these complex arrays. Previous work shows that orienting to faces dominates the attention of both at-risk and control groups of infants'. However, there are substantial individual differences in the degree of preferential orienting towards faces that are larger in the sib-ASD group. Follow-up of these infants at 24-months is currently underway and will be related to diagnostic outcomes.

Non-social attention and orienting is a variant of the "gap task". This task measures the "cost" of disengaging from a central stimulus in order to fixate a peripheral target. In a recent study with infants at-risk[33] reaction time did not differ during baseline trials for the two groups. However, the ASD-sibs group showed a longer reaction time to disengage during overlap trials than did controls.

Neurophysiological assessment of cortical social stimulus processing involves the scalp-recorded ERP and EEG correlates of processing a face compared to another complex stimulus, and a face with direct gaze compared to one with averted gaze, a known biomarker for young children with autism.[34] In previous work with infants at-risk for autism we have shown ERP effects over a posterior channel group.[35] In both infants at-risk and TD controls, the response to face with direct gaze was faster than to averted gaze for two early ERP components (P1 and N290), but infants at-risk showed significantly delayed response to direct gaze but not averted gaze in the later P400 response. Converging results were found using time-frequency analysis, where the control group showed earlier and more clearly differentiated induced gamma activity in response to the direct relative to the averted condition. Consistent with the ERP results, this analysis also indicates that the two groups differ mainly in their processing of the direct gaze condition. We predict that infants at-risk who receive intervention will be restored to the typical pattern of neurophysiological responses to faces observed in low-risk controls, whereas those that do not receive the environmental enhancement will continue to show an atypical pattern characteristic of ASD. For all three biomarkers data from the proposed study will be directly compared to previously collected samples of infants at-risk and controls (N

=50 for each; our UK infant sibs study phase 1) recorded with identical methods and at the same test ages.

2. Are there definable interactional consequences of atypical development in high risk autism siblings?

There are emerging detailed studies of early parent-child interaction in infant siblings at high risk of autism (A-sibs). One study of unstructured play examined the proportion of infant- and mother-led interactions at four months in 21 A-sibs and 21 typically developing (TD)-sibs.[48] During infant-led play, a quarter of A-sibs (and no TD-sibs) exhibited low synchrony, a finding which suggests that their mothers tended to show low affect matching. Three further studies using the structured 'still-face' paradigm (in which the mother interacts with the infant, then freezes on an expressionless face, then resumes interaction) at 4-6 months have also found atypicalities. Compared with TD-sibs, A-sibs showed more neutral affect at typically stressful points (during the still face[48] or at reunion[49]) and smiled less across all episodes[49] and a third of A-sibs showed diminished gaze to the mother's eyes relative to her mouth during the still-face episode compared with 1 in 24 TD-sibs[50] – although looking to the mouth predicted better language outcomes at 24 months.

Our own recent exploratory study[51] is the first to investigate the relationship between specific signs of behavioural atypicality in infancy and details of parent-child interaction. Using blind ratings of global mother-infant interaction, infant siblings of children with autism (6-10 months) with markers indicating potentially increased risk (top quartile scores on the Autism Observation Scale for Infants[22,63] were less attentive to or highly avoidant of their mother during interaction (3/11 or 27%) compared with low risk infants (21/44 or 48%). Mothers of these top quartile AOSI infants showed lower 'sensitive responding' (Fig 1: on a 7-point scale: mean 2.82; SD .98 v mean 3.80; SD 1.41; ANOVA: F=4.70; p=0.04) and low 'acceptance' (mean 2.64; SD 1.21 v mean 3.64; SD 1.57; F=3.86; p=0.055). These findings are consistent with findings from our investigations, at a later developmental age, of parent-child interaction in diagnosed pre-school autistic children. In one study, we used standardised observations of parent-child play in 27 children with core autism (mean age = 46 months, SD = 7.83) compared with data from a group of 24 typically-developing children (mean age = 23 months, SD = 6.50) group-matched on non-verbal ability. We found overall reduced scores on parental 'responsivity' in Autism compared with matched TD controls[52] (p=<.05; fig 2). In a separate study of 27 children with core autism, we found reduced parental communicative interactional synchrony with the autistic child, which was increased following parent-mediated treatment targeted at improving communication[53]. Independent studies in preschool age samples have shown that the degree of such parental synchronous response to the child's focus of attention and activity predicts joint attention and language outcomes in the child many years later.[54]

Taken together these findings suggest that:

1. Relevant aspects of parent-child interaction can be measured during infancy
2. Global measures suggest specific interactional consequences to risk markers of even subtle atypicality in high risk infant sibs within the first year.
3. Such findings are consistent with interactional patterns in diagnosed autistic children
4. These interactional patterns can be modified with targeted intervention.

3. Modelling the intervention strategy

The above rationale implies the need for an intervention strategy that acts on the proximal social environment of the infant (in infancy, parent-child interaction) to best promote developmental trajectories. We will therefore target the intervention on key aspects of parent/child interactive behaviour that are: a) potentially relevant to development in autism and b) show evidence of being amenable to change through intervention. While there is little evidence as yet as to the effect of a prodromal infancy intervention for high risk autism

siblings, our prior work and our review of available evidence suggests candidate targets fulfilling these criteria in a number of areas:

a) Enhancement of parental synchronous responsiveness to infant signals. Our empirical research (section 2 above and[51]) suggests that global parental responsiveness and sensitivity to infant signals may be reduced in association with infant behavioural atypicality in high risk autism siblings. There is a strong evidence base regarding the effectiveness of targeted parent-mediated video-aided interventions in just this area to enhancing parental sensitivity and responsiveness, both in TD and generally 'at risk' infants. A metaanalysis of 81 studies (n=7636[56]), including 51 RCTs (n=6282) of interventions to improve maternal sensitivity found that brief, focussed, personalised video aided intervention with parents was most effective (overall, d=.45). Greater effect was found with relatively briefer treatments focussed in the latter part of the first year (>6 months, d=.44; <6months d=.28). Substantive RCTs of such interventions on high social-risk non-autistic samples using focussed video feedback + education (3 home sessions at 6-9 months[57,58]) have found moderate to large effect sizes on improving maternal sensitivity and child functioning. These intervention strategies provide models of effective interventions in this domain at similar ages in infancy to our study population. They show that intervention can improve parent-child interaction in just the domain we have shown above to be implicated in high risk autism sibs. This evidence gives us the rationale for the core intervention approach in our current study. Furthermore, such a generic approach has the added value of being shown to be appropriate as a universal naturalistic intervention for parental enhancement independent of the risk status of the infant; maximising its ethical legitimacy and acceptability to parents.

However, while there is good evidence that such an approach will enhance generic parental function; the particular characteristics of development in infants at high risk for autism may well necessitate specific additional adaptations. We therefore plan supplementary components of the intervention (described next) which are more autism-specific, although they are of a character that do not either imply nor depend treated infants being prodromal for autism.

b) Parental synchronous response to infant communication. Convergent findings from studies of diagnosed autism in the pre-school period suggest the effectiveness of targeted communication based interventions in altering language communication and other aspects of social functioning. Our own studies in this area[53,59] have contributed to this literature in the UK context. Members of the group are currently undertaking the largest intervention study yet undertaken into the effect of a targeted developmentally informed treatment on pre-school autistic disorder. Studies from our group have shown that a relatively brief parent-mediated video aided intervention can both improve autism symptoms[53] and child communication initiation through enhanced interactional synchrony and individually adapted communication between mother and child.[60] The logic of this evidence, based on enhanced analysis and adaptation of naturally occurring dyadic communication interaction, suggests that appropriate elements of our pre-school intervention may also be adapted into the infancy context. Such communication elements will supplement the generic 'sensitivity training' described above with autism-specific components known to be relevant in pre-school diagnosed autism; and we will be able to test them in the context of high risk sibling samples in infancy.

EXPERIMENTAL PLAN AND METHODS

Design.

A single blinded pilot RCT of two parallel groups: intervention and non-intervention. Research assessments made independently and blind to treatment status at baseline and following 5 months of intervention. Families will be recruited into the study through a recruitment database of the British Autism Study of Infant Siblings, a collaborative research network for the study of infants at-risk for autism (BASIS; REC reference 08/H0718/76). The

design also builds on the ongoing BASIS Phase 1 study (REC reference 06/MRE02/73) where the previously approved experimental protocol will be used for the baseline and post-intervention assessments. The latter protocol has been run with over 100 families taking part in the study based at the Centre for Brain and Cognitive Development.

Recruitment and Consent
Siblings of autism probands will be sampled within the context of a collaborative research network, the British Autism Study of Infant Siblings (BASIS, see attached documentation), and would run in close collaboration with the MRC funded Phase 1 of the UK Baby Siblings programme at the Centre for Brain and Cognitive Development (CBCD), Birkbeck, University of London. Recruitment and ascertainment procedures to the BabyLab at CBCD are well established and ethically approved (see appendices). This gives a robust context within which to undertake an intervention trial in this innovative area for the UK, in terms of infrastructure support, ethics, acceptability to families and procedures. Additionally the trial benefits from incidental lab support (at no cost to the study) and the potential availability of a large body of data collected in the same laboratory on high risk infant siblings and typically developing infants for comparison and generalisation.

Selection criteria
Inclusion criteria: Families sequentially recruited to BASIS that live within therapist travel distance of treatment centres in London and Manchester (south east and north west UK; total population base of approx 14 million). We will not select by extent of phenotypic atypicality. Our rationale for this is: 1) current markers of atypicality are of lower predictive value before 1 year and do not preclude 'false negatives' with respect to autism development; 2) we consider that this increased uncertainty of prediction before the first year makes it ethically most appropriate not to select cases; selection by putative risk would necessitate that we feedback this fact to families, which would be ethically dubious. 3) preserving maximal dimensional variation in atypicality will allow us to examine the impact of intervention in all cases, even those who may not express clear behavioural signs (but may still be different at the level of biomarkers). This will give maximal power to our analysis. In sub-analysis we plan to investigate whether parental enhancement intervention would have better effect on behavioural atypicality as measured by AOSI: such a differential effect of environmental input would be an important finding.
Exclusion criteria: More than one sibling from the same family to preserve independence of subjects within analysis.

Feasibility
Within the 10 month recruitment period of the study, prior data suggests that 120 infant siblings of children with autism age 6-9 months will be recruited into BASIS. These families are recruited largely from self-referral following general advertisement in the media and UK autism networks. Systematic and anonymous feedback using questionnaires collected from families who took part in the CBCD baby siblings programme over the past 3 years has demonstrated that these families are already highly motivated to participate in autism research and recognise the potential broader impact of this infancy research. The target sample for i-BASIS is 50 and thus can be achieved with an opt-in as low as 40%.

Procedures
Randomisation: After research staff have confirmed eligibility and obtained consent, password-protected and encrypted details will be sent to the randomization centre (Christie Hospital Health Care Trials Unit, Manchester). Allocation will be by minimization: controlling for treatment centre (Manchester/London); gender; age band at recruitment (6-7,5 months/7.5-9 months); baseline AOSI score (as variables with potential impact on treatment

response or biomarker expression). The case will be assigned a study number and treatment allocation communicated separately to the treatment centre therapists.
Protection against other sources of bias. Researchers will be housed separately from staff involved in therapy and will attend separate meetings. Baseline assessment will be undertaken prior to parents being informed of treatment assignment. Research interviews will be constructed so as to avoid inadvertent divulging of information that could infer treatment status. The behavioural measure (AOSI) is rated by videotape blind to case details and treatment status. A random 20% of AOSI assessments (20 in total) will be check rated by an external blinded expert; one of the originators of the instrument. The assessment suite and materials used will be quite different in type and location to that used for the treatment intervention avoiding any familiarity effect for children in the treatment arm. Bias due to therapist effects will be minimised by frequent check on continuing therapist fidelity. All treatment sessions are videotaped. 5% of these sessions will be scrutinised by independent clinicians against fidelity criteria in the treatment.

Pre- and post intervention measures
Families taking part in the study will be invited to the babylab in central London. The common BASIS protocol of parent-report and standardised tasks will be administered (please refer to the BASIS research protocol for details). In addition, some experimental tasks will be also administered. These tasks are taken from a previously approved protocol of the "Longitudinal Study of Infant Siblings" (REC reference 06/MRE02/73). The tasks are detailed in the following section. Please refer to the previous research protocol for further details.

Intervention programme
The intervention design derives from a combination of developmental theory and the best current evidence for effective interventions for parent-child interaction in typically developing infants at high and low risk as well as in pre-schoolers with autism (see above). It is parent mediated and video assisted, integrating video-aided techniques to enhance parent-infant interaction.[57,61] Core methods include: (1) A focus on dyadic, communicative aspects of the relationship, with a high degree of adaptation for each particular parent-infant dyad; (2) Videoclip viewing of 'successful' interactions, providing positive examples of sensitive, competent parenting; (3) Involvement of a trained therapist to frame observations to assist parent's self-reflection, and focus discussion and behavioural change. Parents' sense of efficacy is enhanced by active participation and the support given for their intuitive knowledge of their child. Intervention content focusses initially on enhancing parental observation, the attribution of communicative intent to infant behaviours that may be difficult to interpret and facilitating contingent parental responding and affective attunement.[51] To this foundation is added related components manualised and tested in our pre-School autism treatment studies[53]; promoting interactional synchrony and pre-linguistic skills such as joint attention. Our future findings in relation to specific interaction perturbations associated with atypicalities may lead to the inclusion of other specific elements.

The intervention will be manualised and delivered at the family home by specifically trained therapists with a psychiatry, psychology, speech and language therapy or therapeutic nursing/health visitor background. There will be twelve sessions at weekly intervals for three months and a further 4 sessions at two weekly intervals for two months; a total of 16 sessions. There is also 30 mins daily home practice for parent and infant between these sessions. In relation to intensity; less sessions are involved than in our current Pre-School Autism Communication Trial at 2-5 yrs, because we bear in mind the age of subjects and evidence that overlong interventions in infancy may be counterproductive.[56] On the other hand there are more sessions than in the TD infancy interventions reviewed above, bearing in mind the greater potential complexity of these high risk siblings in the context of autism.

Timing of assessments and intervention.

Recruitment will take place between 6-9 months. Intervention will start within 2-4 weeks of baseline. Follow up assessments will be completed within 1 month of treatment ending. Current evidence suggests that risk markers for development of autism become progressively clearer after 12 months. Our intervention is thus targeted at the cusp time at which more predictive risk atypicalities are thought to appear. This timing immediately prior to that therefore in theory maximises the opportunity for plasticity of development and preventative action whilst maximising the likelihood of significant atypicality to work on in the intervention. Furthermore, the timing of assessments coincides with the wider BASIS assessment protocol, resulting in the availability of extensive data on our putative neurophysiological biomarkers and their interactional consequences for comparison and reference and generalisation at the assessment points (see appendix). This timing therefore maximises the reliability of pre- post- measurement of neurophysiological biomarkers and their interactional consequences.

Measures (also see enclosed Summary of Measures document)

A. Generic BASIS measures. (Baseline and follow up (BASIS; REC reference 08/H0718/76).)

In addition to the specific measures related to study hypotheses (as below), participants will receive the core battery of measures common to the BASIS cohort (see appendix 1 for details). These include reference measures of (a) General cognitive and adaptive functioning: and (b) Social-communicative development which will allow group comparison with a larger representative sample. The Development and Wellbeing Assessment (DAWBA)[62] will be used to confirm the diagnosis of the older sibling according to BASIS protocol.

B. Acceptability and impact of intervention.

We will conduct detailed interviews with participating parents before and after the intervention, to gather information on their experience of the treatment and its acceptability. These will include:

1. Pre-Intervention
    i. Parental expectations of the intervention
    ii. General prior parental attitudes towards an intervention of this nature
    iii. Adequacy of information provided prior to delivery of the intervention

2. Post-Intervention
    i. Suitability of the environment in which the intervention was delivered
    ii. Responsivity of and rapport with the therapist
    iii. Acceptability of the sessions with respect to duration and frequency
    iv. Acceptability of the home practice expected between the sessions
    v. Evaluation of whether expectations were met exceeded or unfulfilled, with explanations of why this was the case.
    vi. Components of the intervention that were most useful, with explanations of why this was the case
    vii. Components of the intervention that were least useful, with explanations of why this was the case

3. Wider Impact of Intervention
    i. Consequences of the intervention, both positive and negative, for the relationship between the parent and the infant
    ii. Consequences of the intervention, both positive and negative, for the relationship between the parent and their child with ASD

iii. Consequences of the intervention, both positive and negative, for the relationship between the primary caregivers

C. Behavioural atypicality (BASIS; REC reference 08/H0718/76)**.** The Autism Observation Scale for Infants (AOSI [22, 63]) is a battery of risk markers, focusing on precursors of abilities present in later the autism phenotype; including response to name, eye contact, social reciprocity, and imitation. Additionally the battery examines hypothesised precursor skills such as visual tracking and disengagement of visual attention. Initial data on the instrument at 12 months[22] showed prediction to autism diagnosis at 24 months with a sensitivity of 84% and specificity of 98%, using a 7+ marker threshold. Predictive markers included atypicalities in eye contact, orienting to name, social smiling, and prolonged latency to visual disengagement. Inter-rater reliability both for total scores and number of endorsed items is good to excellent at 6, 12 and 18 months; test-retest reliability is acceptable 12 months. AOSI will be measured at baseline and follow up in a lab setting with trained administrators. Training and reliability support will be undertaken by one of the originators of the instrument.[22]

D. Neurophysiological and saccadic movement biomarkers (baseline and endpoint) (Longitudinal Study of Infant Siblings; REC reference 06/MRE02/73).
Measures will be the same at both timepoints with slight variations in the stimuli to ensure some degree of novelty. All of these tasks have been administered at both ages previously in our lab and continue to be age appropriate.
Social orienting and attention. We will use *eye tracking* to assess several aspects of social orienting, perception and cognition: *Face pop-out*: In this paradigm infants are presented with a series of visual arrays composed of five objects in different spatial locations on a screen. The objects include colourful cars, watches etc, and one photographic image of a face of the same size. Infants watch 12 different arrays each presented for up to 20 seconds. Data on 6-month old infants[32] showed that by several measures (time spent on each object, direction of first look, number of saccades directed to each object) typically developing infants spend proportionately more time on, or repeatedly returning to, the face stimulus. Again, data from the present study can be compared to larger samples of infants at-risk and controls obtained at the same ages with the same procedures. Saccadic responses are analysed based on eye-tracking by means of infra-red detection (Tobii-1750). All visual stimuli used will be designed to be well above acuity and contrast sensitivity thresholds for the age groups concerned. Key measures derived from these and tested pre- and post-intervention are: frequency of saccades and fixation duration (ms) towards a given stimulus, and more specifically to the face relative to competing stimuli averaged across trials.
Non-social attention: *The Gap task* has been studied in detail in infants, children and adults with and without ASD.[2,14] It involves measuring saccadic reaction time to a peripheral visual target, either in the continuing presence of a central fixation stimulus (overlap trials) or with a time gap between the offset of the central fixation stimulus and the onset of the peripheral target. Zwaigenbaum et al.[22] presented preliminary evidence that difficulty in disengaging from a centrally presented visual stimulus could be an early marker for later emerging ASD symptoms, a finding further supported by our own work showing group differences between ASD-sibs and controls at 9 months of age (Elsabbagh et al. submitted, IMFAR 2008). Our procedure uses improved methods from those previously employed, allowing us to also measure baseline and cue facilitation effects. Key measures derived are the reaction time difference between conditions corresponding to disengagement (Baseline vs. Overlap) and facilitation (Baseline vs. Gap) effects.
Electrophysiological measures of cortical social processing:
The ERP paradigm efficiently combines a number of tasks we have previously developed for testing typically developing infants. EEG is recorded as infants view static photographs of female faces displaying direct or averted gaze and analysed using: Event Related Potentials

(ERP) and Time Frequency Analysis of oscillatory brain activity in the gamma band (20-60 Hz). This paradigm compares faces to structured visual noise [64], and gaze shifts moving toward or away from the infant. Thus, this paradigm builds closely on our previous results from young children with autism[34] and typical infants and adults (see[65] for review) but also includes comparisons that will allow us to assess preliminary reports of atypical face and gaze processing in ASD-sibs as a group. ERP procedures and reporting follow established guidelines[66], with a few minor modifications specific to testing infants.[2] We have published direct comparisons between data recorded by the EGI net, and those recorded from conventional low-density systems.[2] With regard to the analysis of ERP data, we will continue to utilise two approaches. The first approach is conventional ERP data analysis in which the main step is finding the temporal coherence of the recorded EEG by averaging time-locked to relevant events (stimulus presentation or action onset). In the resulting ERPs, peaks and deflections can be identified in groups of neighbouring channels, and differences between experimental conditions in the latency or amplitude of these "components" are statistically evaluated. It is important to note that the presence or absence of differences between experimental conditions can be identified even if there are differences in the overall waveform between age groups or clinical populations.

E. Parent Child Interaction (BASIS; REC reference 08/H0718/76)**.**
A standard five-minute unstructured play paradigm will be employed, in which the mother is instructed to play with their infant as they would normally (on a floor mat, with or without toys). The videotaped interactions will be coded independently blind to risk status using a 7-point global rating scale, which has been modified from validated measures to suit our study group by combining the brevity/age appropriateness of the Global Rating Scales of Mother-Infant Interaction (GRS[4]) and the autism-specificity and format of the 'Coding of Attachment-Related Parenting -for use with children with Autism' (CARP[67]). The new scale, which has demonstrated feasibility and inter-rater reliability[51] comprises 3 maternal items (sensitive responsiveness, acceptance, appropriate affect), 3 infant items (attentiveness /avoidance, appropriate affect, positive vocalisation), and 2 dyadic items (synchrony/mutuality, quality of engagement). This will be measured using the standard 5 minutes free play paradigm between parent and infant video taped for blind coding. Coding will be undertaken on generic, language-specific, and atypicality-specific dimensions using adaptations of the CARP and GRS measures.

DATA ANALYSIS

General Issues:

Intervention and Developmental Analysis: The general approach to analysis will span the more pre-specified formula of RCT reporting of the intervention effect, for which the study has been powered, and the more exploratory developmental analysis within which we will be viewing the intervention as a randomised experimental manipulation of the social environment. In light of the sensitivity of measures to maturation all analyses will covary for age.
Data reduction: In general, standard scoring schemes and cut-offs will be used wherever possible. Systematic data cleaning and checking will be undertaken. Pro-rating of occasional missing items will be used in the calculation of item total scores.
Missing Data: Analyses of data involving missing measures will be undertaken wherever possible either by maximum-likelihood or by the use of multiple imputation, carried out using the iterative chained equation approach as implemented in Stata [68]. Both approaches allow for potentially selective but non-informative attrition.
Effect Estimators: Analysis of the intervention effect element of the study will initially use an intention to treat (ITT) analysis to compare endpoint group effects using apriori hypotheses related to each measurement domain. Where non-compliance is a concern or parent training proves to be of variable success, alternative effect estimates will be calculated, such as the

Local Average Treatment Effect.[18] These continue to exploit the randomised treatment allocation undertaken, and are also valuable in obtaining estimates of mediation unbiased by residual confounding.

Significance and Precision: In the light of the modest sample size and concern about the appropriateness of asymptotic estimators of precision and significance, bootstrap methods will be used.

Analysis of Behavioral Measures

The behavioural measures analysis will focus in the first instance on the simple total markers derived from the AOSI. We will make use of the structural equation modelling set up that is equivalent to Analysis of Covariance. This allows the addition of age at assessment as an additional covariate to both baseline and endpoint scores.

<u>Hypothesis</u>: the intervention will reduce the total specific precursor markers for ASD.

Towards the end of the study information will have accumulated follow up data to enable more confident assessment of the degree of any ASD symptomatology. We will then use signal detection methods to estimate and refine the performance of early biological and cognitive markers against available current best estimates of ASD as data is acquired. In view of the modest sample size leave-one-out cross-validation methods will be used. Given current estimate of recurrence rates, the sample size of this study will not have the power to test definitively the effect of infancy intervention on eventual diagnostic autism outcome. However a positive result from this study would support larger sample studies over a longer time period to do this.

In a number of areas of skill, development is characterised by the timed achievement of milestones. Available data is often interval censored, indicating a window of time in which a milestone was achieved, for example from repeated questionnaires on current status (as in the Vineland) or imperfect recall (as in the ADI-R). We will use multivariate interval censored survival methods to assess the effects of the intervention in hastening skill acquisition/normative development or delaying onset of ASD characteristic symptoms.

Parent-child interaction measurement is our hypothesised mediator of the treatment effect. Dimensional summary measures from the coding of interaction with the sibs will be analysed using MANOVA and Structural Equation Models to test and examine the structure of the association among these variables at baseline, the structure of the pattern of association in their change over time, the difference according to risk group, and the differences associated with the intervention.

Saccadic movement and Neurophysiological analysis. The logic of this overall design is that we will be able to compare (1) pre- and post intervention measures, (2) developmental changes in the no intervention group, and (3) the two groups at the endpoint. In addition to comparisons with our substantial existing data set on these same tasks (UK infant sibs phase 1, which will include FU to diagnosis during the course of this study), we will thus be able to describe the typical developmental trajectory in these measures, the at-risk infant (broader phenotype) trajectory, and the effects of our environmental enhancement on the at-risk infant trajectory. Analyses will test for group by condition interactions, controlling for age through parametric analysis using general linear models.

<u>Hypotheses:</u>

Face pop-out: Levels of pre-intervention fixation duration towards the face will increase relative to distactors in the experimental group but not in the control group.

Gap task: controlling for the effects of age, there will be shorter latencies to disengage in the overlap condition post-intervention relative to pre-intervention in the experimental group relative to the control group, but there would be no change in the baseline condition. The disengagement effect post-intervention would be smaller in the intervention group relative to the control group.

ERP task: controlling for the effects of age there will be shorter latencies in the P400 component in response to direct gaze in the experimental group relative to the control group. On the other hand, there would be no change in the response to the averted gaze condition.

In secondary exploratory analysis we will also be able to test general task parameters (e.g., response in early visual ERP components and overall levels of orienting in saccadic reaction time tasks) to examine whether more general effects can also be observed in the experimental relative to the control group. Additional analyses will examine the extent to which neurophysiological measures and biomarkers are associated with each other and with change in brain function.

Sample size and power

Behavioural markers. The proposed pilot two-group (n=25 per group) intervention study would have a nominal power of 80% (two-tailed alpha=0.05) for an outcome group difference of 0.8SD on the behavioural phenotype measure (AOSI); slightly smaller than that we have found in our intervention for diagnosed children.[53]

Saccadic movement and Neurophysiological analysis. Gap task Disengagement effect: GLM with condition as within (Baseline, Overlap) and Group as between (Sib-ASD vs. Control). N=15 sib-ASD and 16 ctl yields effect size (partial eta squared) = 0.17 and observed power of 0.66 (at alpha=0.05). Gaze ERP latency of P400 component: GLM with condition as within (Direct, Averted) and Group as between (Sib-ASD vs. Control). N=17 sib-ASD and 19 ctl yields effect size (partial eta squared)=0.16 and observed power of 0.70 (at alpha=0.05). We can be therefore confident that the N of 25 in each group in this study will substantially exceed this in power.

Timeline

Month	Activity	Personnel	Milestones/output
1-4	Start up	Research assessors (RA)<br>Research Treatment Therapists (RTT)	Training of RAs in assessment methods<br>Training of RTTs
4-24	Recruitment, consenting	RAs	Recruitment of minimum N=50 subjects into randomised trial by Month 24 (= 2.5 subjects/month)
5-26	Baseline assessments	RAs	
7-29	Treatment intervention	RTTs	Each treatment cycle @ 5 months. Total of n=25 treatments minimum over 23 months
10-30	Follow up assessment	RAs	
7-30	Ongoing coding/processing of neurophysiological data	RA (Birkbeck), PIs (MJ,ME)	
7-30	Ongoing coding of parent	RA (Manchester),	

	child interaction data	PIs (MWW, JG, VS)	
31-34	Data analysis	RA (Statistics), PI (AP)	
33-36	Report preparation	PIs	

REFERENCES

1. Johnson, M.H., *Functional brain development in humans.* Nature Reviews Neuroscience, 2001. **2**(7): p. 475-483.
2. Johnson, M.H., H. Halit, S.J. Grice, and A. Karmiloff-Smith, *Neuroimaging of typical and atypical development: A perspective from multiple levels of analysis.* Development and Psychopathology, 2002. **14**(3): p. 521-536.
3. Dunham, P. and F. Dunham, *Effects of Mother-Infant Social Interactions on Infants Subsequent Contingency Task-Performance.* Child Development, 1990. **61**(3): p. 785-793.
4. Murray, L., A. FioriCowley, R. Hooper, and P. Cooper, *The impact of postnatal depression and associated adversity on early mother-infant interactions and later infant outcome.* Child Development, 1996. **67**(5): p. 2512-2526.
5. Watson, J.S., *Contingency perception in early social development.*, in *Social perception in Infants*, T.M. Field and N.A. Fox, Editors. 1985, Ablex: New Jersey. p. 157-175.
6. Trevarthen, C., *Conversations with a two-month old.*, in *New Scientist*. 1974. p. 230-235.
7. Cardosomartins, C. and C.B. Mervis, *Maternal Speech to Prelinguistic Children with Down Syndrome.* American Journal of Mental Deficiency, 1985. **89**(5): p. 451-458.
8. Crawley, S.B. and D. Spiker, *Mother-Child Interactions Involving 2-Year-Olds with Down Syndrome - a Look at Individual-Differences.* Child Development, 1983. **54**(5): p. 1312-1323.
9. Sorce, J.F. and R.N. Emde, *The Meaning of Infant Emotional Expressions - Regularities in Caregiving Responses in Normal and Downs-Syndrome Infants.* Journal of Child Psychology and Psychiatry and Allied Disciplines, 1982. **23**(2): p. 145-158.
10. Dunst, C.J., *Communicative competence and deficits: effects on early social interactions*, in *Facilitating social-emotional development in multiply handicapped children. Philidelphia Home of the Merciful Saviour for Crippled Children.*, E.T. McDonald and D.L. Gallagher, Editors. 1985: Philidelphia. p. 93-140.
11. Slonims, V., A. Cox, and H. McConachie, *Analysis of mother-infant interaction in infants with Down syndrome and typically developing infants.* American Journal on Mental Retardation, 2006. **111**(4): p. 273-289.
12. McCathren, R.B., P.J. Yoder, and S.F. Warren, *The Role of Directives in Early Language Intervention.* Journal of Early Intervention, 1995. **19**(2): p. 91-101.
13. Harris, S., C. Kasari, and M.D. Sigman, *Joint attention and language gains in children with Down syndrome.* American Journal on Mental Retardation, 1996. **100**(6): p. 608-619.
14. Landry, R. and S.E. Bryson, *Impaired disengagement of attention in young children with autism.* Journal of Child Psychology and Psychiatry, 2004. **45**(6): p. 1115-1122.
15. Legerstee, M., J. Varghese, and Y. van Beek, *Effects of maintaining and redirecting infant attention on the production of referential communication in infants with and without Down syndrome.* Journal of Child Language, 2002. **29**(1): p. 23-48.

16. Yoder, P.J. and S.F. Warren, *Early predictors of language in children with and without Down syndrome.* American Journal on Mental Retardation, 2004. **109**(4): p. 285-300.
17. Walden, T.A., J.U. Blackford, and K.L. Carpenter, *Differences in social signals produced by children with developmental delays of differing etiologies.* American Journal on Mental Retardation, 1997. **102**(3): p. 292-305.
18. Green, J. and G. Dunn, *Using intervention trials in developmental psychiatry to illuminate basic science.* British Journal of Psychiatry, 2008. **192**(5): p. 323-325.
19. Howe, G.W., D. Reiss, and J. Yuh, *Can prevention trials test theories of etiology?* Development and Psychopathology, 2002. **14**(4): p. 673-694.
20. Elsabbagh, M. and M.H. Johnson, *Infancy and autism: progress, prospects, and challenges*, in *Progress in Brain Research*, v.H. C. and Rosander, Editors. In press, Elsevier. p. 355-383.
21. McCleery, J.P., E. Allman, L.J. Carver, and K.R. Dobkins, *Abnormal magnocellular in infants at risk for autism.* Biological Psychiatry, 2007. **62**(9): p. 1007-1014.
22. Zwaigenbaum, L., S. Bryson, T. Rogers, W. Roberts, J. Brian, and P. Szatmari, *Behavioral manifestations of autism in the first year of life.* International Journal of Developmental Neuroscience, 2005. **23**(2-3): p. 143-152.
23. Nadig, A.S., S. Ozonoff, G.S. Young, A. Rozga, M. Sigman, and S.J. Rogers, *A prospective study of response to name in infants at risk for autism.* Archives of Pediatrics & Adolescent Medicine, 2007. **161**(4): p. 378-383.
24. Landa, R.J., K.C. Holman, and E. Garrett-Mayer, *Social and communication development in toddlers with early and later diagnosis of autism spectrum disorders.* Archives of General Psychiatry, 2007. **64**(7): p. 853-864.
25. Dawson, G., S. Webb, G.D. Schellenberg, S. Dager, S. Friedman, E. Aylward, and T. Richards, *Defining the broader phenotype of autism: Genetic, brain, and behavioral perspectives.* Development and Psychopathology, 2002. **14**(3): p. 581-611.
26. BaronCohen, S. and J. Hammer, *Parents of children with Asperger Syndrome: What is the cognitive phenotype?* Journal of Cognitive Neuroscience, 1997. **9**(4): p. 548-554.
27. Ozonoff, S., S.J. Rogers, J.M. Farnham, and B.F. Pennington, *Can Standard Measures Identify Subclinical Markers of Autism.* Journal of Autism and Developmental Disorders, 1993. **23**(3): p. 429-441.
28. Hughes, C., M. Leboyer, and M. Bouvard, *Executive function in parents of children with autism.* Psychological Medicine, 1997. **27**(1): p. 209-220.
29. Happe, F., J. Briskman, and U. Frith, *Exploring the cognitive phenotype of autism: Weak "central coherence" in parents and siblings of children with autism: I. Experimental tests.* Journal of Child Psychology and Psychiatry, 2001. **42**(3): p. 299-307.
30. Rueda, M.R., M.K. Rothbart, B.D. McCandliss, L. Saccomanno, and M.I. Posner, *Training, maturation, and genetic influences on the development of executive attention.* Proceedings of the National Academy of Sciences of the United States of America, 2005. **102**(41): p. 14931-14936.
31. de Haan, M., J. Belsky, V. Reid, A. Volein, and M.H. Johnson, *Maternal personality and infants" neural and visual responsivity to facial expressions of emotion.* Journal of Child Psychology and Psychiatry, 2004. **45**(7): p. 1209-1218.
32. Garwood, H., J. Belsky, M. Elsabbagh, A. volein, L. Tucker, S. Baron-Cohen, P. Bolton, T. Charman, G. Baird, and M.H. Johnson. *Orientatinig to social and non-social stimuli in the early broader autism phenotype.* in *IMFAR*. 2008. London, UK.
33. Elsabbagh, M., A. volein, K. Holmboe, L. Tucker, G. Csibra, S. Baron-Cohen, P. Bolton, T. Charman, G. Baird, and M.H. Johnson. *Visual orientating in infants at risk for autism.* in *IMFAR*. 2008. London, UK.
34. Grice, S.J., H. Halit, T. Farroni, S. Baron-Cohen, P. Bolton, and M.H. Johnson, *Neural correlates of eye-gaze detection in young children with autism.* Cortex, 2005. **41**(3): p. 342-353.

35. Elsabbagh, M., A. volein, K. Holmboe, L. Tucker, G. Csibra, S. Baron-Cohen, P. Bolton, T. Charman, G. Baird, and M.H. Johnson. *Nerual correlates of eye-gaze processing in the early autism phenotype*. in *IMFAR*. 2007. Sealtte, USA.
48. Yirmiya, N., I. Gamliel, T. Pilowsky, R. Feldman, S. Baron-Cohen, and M. Sigman, *The development of siblings of children with autism at 4 and 14 months: social engagement, communication, and cognition.* Journal of Child Psychology and Psychiatry, 2006. **47**(5): p. 511-523.
49. Cassel, T.D., D.S. Messinger, L.V. Ibanez, J.D. Haltigan, S.I. Acosta, and A.C. Buchman, *Early social and emotional communication in the infant siblings of children with autism spectrum disorders: An examination of the broad phenotype.* Journal of Autism and Developmental Disorders, 2007. **37**(1): p. 122-132.
50. Merin, N., G.S. Young, S. Ozonoff, and S.J. Rogers, *Visual fixation patterns during reciprocal social interaction distinguish a subgroup of 6-month-old infants at-risk for autism from comparison infants.* Journal of Autism and Developmental Disorders, 2007. **37**(1): p. 108-121.
51. Wan, M.W., J. Green, M. Elsabbagh, and M.H. Johnson, *Mother-infant interactions in high-risk infant siblings of children with autism.*, in *International Meeting for Autism Research*. 2008: London, UK.
52. Blazey, L., K. Leadbitter, C. Holt, and J. Green. *Attachment behaviours and parent-child interation in pre-school autism*. in *IMFAR*. 2008. London UK.
53. Aldred, C., J. Green, and C. Adams, *A new social communication intervention for children with autism: pilot randomised controlled treatment study suggesting effectiveness.* Journal of Child Psychology and Psychiatry, 2004. **45**(8): p. 1420-1430.
54. Siller, M. and M. Sigman, *The behaviors of parents of children with autism predict the subsequent development of their children's communication.* Journal of Autism and Developmental Disorders, 2002. **32**(2): p. 77-89.
55. Green, J., M.W. Wan, and M. Deklyen, *Attachment insecurity and attachment disorder*, in *Cambridge Textbook of Effective Treatments in Psychiatry*, P. Tyrer and K.R. Silk, Editors. 2008, Cambridge University Press: Cambridge. p. 748-755.
56. Bakermans-Kranenburg, M.J., M.H. van Ijzendoorn, and F. Juffer, *Less is more: Meta-analyses of sensitivity and attachment interventions in early childhood.* Psychological Bulletin, 2003. **129**(2): p. 195-215.
57. Juffer, F., M.J. Bakerman-Kranenburg, and M.J. Van Ijzendoorm, *Promoting Positive Parenting: An Attachment-Based Intervention.* 2008, New York: Taylor Francis Group.
58. Vandenboom, D.C., *The Influence of Temperament and Mothering on Attachment and Exploration - an Experimental Manipulation of Sensitive Responsiveness among Lower-Class Mothers with Irritable Infants.* Child Development, 1994. **65**(5): p. 1457-1477.
59. Drew, A., G. Baird, S. Baron-Cohen, A. Cox, V. Slonims, S. Wheelwright, J. Swettenham, B. Berry, and T. Charman, *A pilot randomised control trial of a parent training intervention for pre-school children with autism - Preliminary findings and methodological challenges.* European Child & Adolescent Psychiatry, 2002. **11**(6): p. 266-272.
60. Aldred, C., J. Green, and H. McConachie. *Measuring change in parent child communicative interaction during pre-school treatment for autism*. in *IMFAR*. 2008. London, UK.
61. Wels, P.M.A., *Measuring the effects of Video Home Training.*, in *the Fifth EURSARF Congress*. 1995: London, UK.
62. Goodman, R., T. Ford, H. Richards, R. Gatward, and H. Meltzer, *The Development and Well-Being Assessment: Description and initial validation of an integrated assessment of child and adolescent psychopathology.* Journal of Child Psychology and Psychiatry and Allied Disciplines, 2000. **41**(5): p. 645-655.

63. Bryson, S.E., L. Zwaigenbaum, C. McDermott, V. Rombough, and J. Brian, *The autism observation scale for infants: Scale development and reliability data.* Journal of Autism and Developmental Disorders, 2008. **38**(4): p. 731-738.
64. Halit, H., G. Csibra, A. Volein, and M.H. Johnson, *Face-sensitive cortical processing in early infancy.* Journal of Child Psychology and Psychiatry, 2004. **45**(7): p. 1228-1234.
65. Grossmann, T. and M.H. Johnson, *The development of the social brain in human infancy.* European Journal of Neuroscience, 2007. **25**(4): p. 909-919.
66. Picton, T.W., S. Bentin, P. Berg, E. Donchin, S.A. Hillyard, R. Johnson, G.A. Miller, W. Ritter, D.S. Ruchkin, M.D. Rugg, and M.J. Taylor, *Guidelines for using human event-related potentials to study cognition: Recording standards and publication criteria.* Psychophysiology, 2000. **37**(2): p. 127-152.
67. Blazey, L., *Attachment in autism: An investigation into parental sensitivity, mutuality and affect.* 2007, The University of Manchester
68. Royston, P., *Multiple Imputation of missing values.* Stata Journal, 2004. **4**(3): p. 227-234.

INTERVENTION PROTOCOL*

AUTHORS

Samina Holsgrove[1], Ming Wai Wan[1], Janet McNally[1], Rhonda Booth[2], Carol Taylor[1],Vicky Slonims[2], Jonathan Green[1]

1. **University of Manchester**
2. **Evelina London Children's Hospital /Kings College London**

ACKNOWLEDGEMENT

Much of the procedural manual contained here is based on or adapted from the original work and manual of the Video Interaction to Promote Positive Parenting (VIPP) Juffer, F., M.J. Bakerman-Kranenburg, and M.J. Van Ijzendoorm, *Promoting Positive Parenting: An Attachment-Based Intervention*. 2008, New York: Taylor Francis www.leidenattachmentresearchprogram.eu/vipp/welcome/en/

*The full procedural manual is available separately from the authors

AIM

Enrichment of the social interactive environment of the developing infant using a parent-mediated programme to enhance early social engagement and reciprocity.

The intervention strategy

The i-BASIS intervention strategy is a parent-mediated approach to achieve two goals;

1) general enrichment of the core interactive social experience for infants 9-14 months inclusive
2) specific attention within this to addressing any emerging atypicalities that might be expected in prodromal autism at this age and their interactional consequences

The *i*-BASIS programme comprises up to twelve home based two hourly sessions over a period of 5 months. The programme is individualised to the needs of each dyad but core procedures are taken from the Video feedback Intervention to promote Positive Parenting (VIPP)[1], www.leidenattachmentresearchprogram.eu/vipp/welcome/en/). We chose this as the basis because its method (video-aided and parent-mediated using a direct work with parent and infant) is similar to that which we have used intensively with preschool children with diagnosed autism[2] (www.manchester.ac.uk/medicine/pact) and because of its good evidence base across disorders and in neurotypical groups.

There is a preliminary session (baseline/relationship building with parent); followed by six intervention sessions (delivered weekly to fortnightly); each with a theme building on techniques and learning from the previous session. The first to intervention sessions focus on infant behaviour (with maternal behaviour alluded to only indirectly), the third and fourth sessions address maternal behaviour, and the final two sessions examine more complex chains of social interaction. The set up of each session is designed to facilitate exploration of these specific targeted themes. These are followed by up to five booster sessions to consolidate

learning; the number and content of these booster sessions is agreed between therapist and parent based on progress and development in the six themed sessions. Feasibility and acceptability of the method was shown in an independent case series study.[3]

1.Juffer, F., M.J. Bakerman-Kranenburg, and M.J. Van Ijzendoorm, *Promoting Positive Parenting: An Attachment-Based Intervention*. 2008, New York: Taylor Francis Group.

2. Green J. Charman, T,. McConachie, H., Aldred, C., Slomins, V., Howlin, P., Le Couteur, A., Leadbitter, K., Hudrey, K., Byford, S., Barrett, B., Temple, K., MacDonald, W., Pickles, A., and the PACT consortium. (2010). Parent-Mediated Communication-Focused Treatment for preschool children with Autism (PACT); a randomised controlled trial. *The Lancet,* 375(9732), 2152-2160.

3. Green J., Wan MW., Guiraud J., Holsgrove S., McNally J., Slonims V., Elsabbagh M., Charman T., Pickles A., Johnson M., and the BASIS team (2013) Intervention for Infants at Risk of Developing Autism: A Case Series. *J of Autism and Dev Disorders* 43(11):2502-2514.

SESSION PLANS

INTRODUCTORY SESSION

**Aim:**<br>Introduction, rapport building, goal-setting, and baseline measurement.	**Record video for session 1**<br>Six minute parent-infant interaction: free play with toys

SESSION 1 – “INFANT WATCHING”

Theoretical Focus: Sensitive responding

The parent has an opportunity to observe the focus and choice of activity of their infant without interruption. This encourages her to observe their child closely and to recognize the pace of the infant’s exploratory behaviours and to match her own responses accordingly. The parent’s experience of watching her infant may also encourage her to think of him or her as a “thinking” being and help her appreciate the potential positive impact of a timely and sensitive response to her child’s behaviours.

**Aim:**<br>Observing and naming infant social interactive behaviour	**Record video for session 2**<br>Free play interaction (6 mins)<br>Non-interactive play (2 mins)

SESSION 2: ‘ SPEAKING FOR THE BABY’

Theoretical Focus: Inference of intentionality

The observations made of the first session are discussed in depth with a focus on the attribution of intentionality to the infant. The purpose is to reinforce parental empathy with the infant’s affect state as this forms the basis of a sensitive contingent response. The parent is

asked to describe the baby's activities and thoughts speak 'for the child' thus demonstrating awareness of their perspective.

**Aim:**<br>Observing infant interactive behaviour in conjunction with exploratory behaviour	**Record video for session 3**<br>Free play interaction (6 mins)

SESSION 3: 'SENSITIVITY CHAINS'

Theoretical Focus: Synchrony and contingent responsiveness

Building on the concepts introduced in session 2, the parent is encouraged to respond to a range of infant behaviours and match her responses to the behaviour of the infant, thereby increasing synchrony. The identification of sensitivity chains (infant behaviour-maternal response-positive infant response) reinforces the parent's awareness of contingent responsiveness as she demonstrates attunement to her infant's needs.

**Aim:**<br>Encourage parental contingent responsiveness<br>Particular reinforcement of inter-personal face to face type interactions	**Record video for session 4**<br>Naturalistic setting of a meal time or snack time (20-30 mins)

SESSION 4: SENSITIVITY CHAINS AT MEALTIMES

Theoretical focus: Contingent responsiveness in everyday situations

This session focuses on generalizing the skills addressed in session 3 to an everyday context in a naturalistic setting to help the parent integrate skills such as attunement and synchrony with her infant to every interaction between them.

**Aim:**<br>Generalising contingent responsiveness to a naturalistic setting	**Record video for session 5**<br>Face-to-face 'songs and rhymes' interaction (6 mins)

SESSION 5: 'SHARING FEELINGS'

Theoretical Basis: Affect matching

Session 5 introduces a technique to enhance maternal empathy: inviting the parent to speak as if she herself were the infant (subtly different from describing the baby's ideas in session 2). This is carried out using a video clip of face-to-face interaction to encourage affect matching.

**Aim:**	**Record video for session 6**

Encourage affect matching and empathy<br>Reinforcement of inter-personal interactions, including eye contact	Free play with toys, to include reading a book together if possible (4 mins)<br>“Funny Sound Game” (2 mins)

SESSION 6: ”SHARING TALK”

Theoretical Focus: Communication

In this session the mother is assisted to reflect on more subtle aspects of vocal and non-vocal communication in the context of a structured interaction involving book reading. The aim is to support reciprocal vocalisations in a social context with contingent, attuned responses from the parent.

**Aim:**	**Record video for booster session**
Encourage vocal communication and social babble<br>Reinforcement of interpersonal interactions, including eye contact	Free play with toys (6 mins)

SESSIONS 7 – 12: REINFORCEMENT AND BOOSTER SESSIONS; FURTHER MANAGEMENT OF ATYPICALITY

Reinforcement and booster sessions

The aim of these sessions is to reinforce the parent’s learning and ensure progress in parent-infant synchrony, attunement and communication as the infant rapidly learns new skills. This will sometimes involve a return to earlier themes e.g. ‘infant watching’, observation and sensitivity to the infant’s particular traits and reinforcing synchronous responses.

Identification of atypicality

The therapist in the *i*-BASIS study will not have been involved in the baseline assessment. However, during the intervention sessions there will have been adequate time for the therapist to identify any evidence of atypicality in the infant within the therapy context. Therapists use a checklist of potential atypicalitiesas an aide memoire at the end of sessions and rate behaviours on a 0-2 rating scale after the introductory session, and then after the 3rd, 6th, 9th and final sessions. *Identified atypicalities are discussed with the parent in terms of the infant’s behavioural repertoire without labelling them as prodromal signs*. They are identified as potential barriers to the processes of reciprocity and shared communication and appropriate advice is given to facilitate interaction. The degree of interactional perturbation is likely to vary considerably with each infant and parent. Detailed intervention approaches to address potential atypicalities are described in the full procedural manual; the selection of approaches is tailored to the individual dyad. The therapist adopts a collaborative and exploratory approach with the parent to reduce the impact of these potentially atypical behaviours.

We have considered it important to have an intervention that does not assume atypicality in a group of infant siblings of children with autism spectrum disorder. In cases where a parent and infant have successfully established reciprocal and mutually satisfactory interaction within the 6 intervention sessions or before the end of the booster sessions the final visits can be

spaced more or the total number limited by mutual agreement with parent. In this way *i*-BASIS has built-in flexibility to the heterogeneity of development in the intervention group. The generic parental enhancement techniques in VIPP have demonstrated applicability across a range of normative parenting styles; the additional components more specific to prodromal autism can adapt the intervention where children are presenting with differences in development.

I-BASIS INTERVENTION FIDELITY RATING SCALE

Fidelity Coder:

Therapist: Participant ID:

Feedback Session Date: Play Session Date:

Intervention Session Number: 1 2 3 4 5 6 Booster 7 8 9 10 11 12 [circle one]

Material to accompany Rating Scale:

Video of Play Session
Video/audio recording of Feedback Session
Copy of Script
Any relevant info from therapist's Session record for (i) Play or (ii) Feedback Session

A. General Therapeutic Procedures

1. Review of Previous Session including discussion of Practice Tasks (Session 2 onwards)

0 The therapist did not review previous session with parent, including a review of practice tasks.

1 The therapist provides a summary of previous session content and asks how the parent got on with the practice tasks, including whether they had the opportunity to watch the video during the week.

2. Introduction of Current Session Theme

0 The therapist did not introduce the session theme before reviewing the video.

1 The therapist effectively introduces the appropriate session theme (see Appendix) before reviewing the video.

3. Session Theme Illustrated during Video Review

0 The therapist did not illustrate the relevant theme or made comments that were vague or not related to the session theme.

1 The therapist effectively illustrates the session theme (see Appendix), including commenting on the appropriate aspects of the infant's behaviour.

4. Eliciting Parent Feedback

0 Little or no attempt by the therapist to elicit feedback to determine if the parent had understood the strategies and techniques being utilised in the session. Did not ask enough questions to be sure the parent understood the session theme or to ascertain the parent's reactions to the session.

1 The therapist elicited feedback from the parent to determine the parent's observation and understanding of session theme.

5. Response to Parents Focus

0 No attempt to recognise or respond to parents focus.

1 Recognised and responded appropriately to parents focus throughout the session.

6. Managing the Feedback

0 Little or no structure to the feedback time *or* pacing too slow or too fast, or was inflexible or not adapted to the task in hand.

1 The therapist structured the feedback well, so there was a clear beginning, middle and closing of the feedback. Peripheral and unproductive digressions were either very uncommon, or handled well by the therapist.

7. Pacing

0 The therapist pacing and timing was not appropriate to the parents.

1 The therapist pacing and timing was appropriate to the parents.

8. Summary and Setting of Practice Tasks

.

0 The therapist did not provide a summary for the session, and failed to set practice tasks, *or* set tasks that were vague, incomplete, or unilaterally determined.

1 A summary of the main observations from the session was provided verbally by the therapist and on paper. There was setting of practice tasks that arose directly from the session and was jointly agreed.

B. Interpersonal Effectiveness

1. ***Sensitivity Skills***

0 The therapist failed to reflect or rephrase what the parent explicitly said or showed problems responding to implicit or subtle communication.

1 The therapist generally seemed to grasp the parent's meaning as reflected by both what the parent explicitly said and what the parent communicated more subtly.

2. Validation and Positive Feedback

0 The therapist missed opportunities to praise parental achievements.
1 The therapist recognised and appropriately praised parental achievements

C. Specific Criteria

1 The therapist made a positive comment about the interaction or the play situation within the first 10-15 seconds of the video.
Yes/No

2 The therapist made at least one compliment during the feedback; for example, an appropriate and timely parental response (i.e., when the parent responds promptly to cues from her infant).
Yes/No

3 The therapist makes a comment using "Speaking for the baby" at least every 30 seconds.
Yes/No

4 ***From session 3 onwards only:*** Therapist uses 2 sensitivity chains appropriately

Yes/No/NA

5 ***From session 3 onwards only:*** Therapist uses 1 corrective message appropriately

Yes/No/NA

D. Use of Booster Sessions

0 No clear aim established for the booster session.
1 A clear aim to the booster session was established. This may be from previous discussion with parent about which Session theme(s) they would like to repeat, or address any current issues.

Yes/No/NA

E. Deviation from the Manual

Were there any significant unusual factors in this session that you feel justified the therapist's departure from the manual?
Yes/No/NA
Please explain below:

F. Materials

For standardisation of the first and final session: the core play materials were available to the parent and infant's toys removed/minimised.

Yes/No/NA

G. Room Environment

Were distractions minimised; for example, presence of other children, family members, etc or external noises such as TV, radios?

Yes/No

H. Video and Sound

Was the quality of the video and sound adequate? For example, could the facial expressions and interactions between the parent and infant be viewed? Were the sounds made by infant and parent audible?

Yes/No

I. Quality Time

Was there adequate opportunity for parent/therapist discussion?

Yes/No

Appendix: Specific Intervention Techniques for each Therapy Session

Session 1 Infant Watching

Theoretical Focus: Sensitive responding

Observation of the infant's attentional transitions without interruption encourages the parent to recognize the pace of the infant's exploratory behaviours and to match her own responses to this pace. The parent's experience of watching her own infant may also encourage her to think of him or her as a mentalistic being and help her to begin to appreciate the potential positive impact of a timely and sensitive response to her child's behaviours.

The therapist makes the parent aware of their infant's social interactive behaviour by describing the infant's thoughts, emotions and behaviours (1 Speaking for the baby); drawing attention to infant's natural pace (2 Adjusting to the infant's pace); highlighting instances where the infant is allowed to take the initiative (3 Following the infant); and commenting on the mood of the infant (4 Empathising with the infant). Comments made are directed towards the infant's behaviour and response; no comments are made on the parent's behaviour.

Session 2 Speaking for the Baby

Theoretical Focus: Inference of intentionality

The techniques used here build on the observations of the previous session and focus on the endowment of intentionality to the infant. The purpose is to reinforce the importance of parental empathy with the infant's affect state, as this forms the basis of a sensitive

contingent response. From these principles the parent can display this understanding back to the infant, "feeling for them", meaning that the infant feels understood.

The therapist makes the parent aware of any differences in infant behaviour while the parent is playing alongside, versus when the parent stops interacting for two minutes. Emphasis is made on how the infant explores the environment and their capacity to discover and learn on their own compared to when a parent is present.

Some discussion may be made on "attachment behaviours" in the infant, and their need for a secure parental base, and/or sharing interest with a parent. Note that if the infant is very much in an "object focus" stage, any changes in behaviour during the two minute non-interaction phase may not be seen.

In addition to describing infant behaviour (using 1 to 4 described above), the therapist encourages parent to provide subtitles for infant behaviour through questioning; for example, *What do you think she is feeling there?* (5 Speaking for the baby by the parent.) Again, no comments are made on parental behaviour.

Session 3 Responding to each other

Theoretical Focus: *Synchrony and contingent responsiveness*

This session encourages the parent to respond to a range of infant behaviours through matching her own responses to that of the infant, thereby increasing synchrony. This builds on the concept introduced in session 2. The sensitivity chain puts this into practice and reinforces to the parent that through contingent responsiveness she demonstrates attunement to her infant's needs.

The therapist makes effective use of (6) Sensitivity Chains (provides at least two), where positive behaviours of the infant are pointed out that followed a contingent parental response (i.e., parent responds appropriately to a cue from the infant, and then receives a positive response from the infant). The therapist provides at least one (7) Corrective Message: a feedback comment on parental behaviour, that is a focus for change, framed positively, and as a suggestion (e.g., "*Here you could have also done..." "I wonder what would have happen if you had done..." "He really enjoyed that, maybe he would like to do it again?"* Methods, described 1 to 5 above, are also used effectively by the therapist.

Session 4 Responding to each other during normal routines

Theoretical focus: *Contingent responsiveness in everyday situations*

This session focuses on generalizing the skills utilized in session 3 to an everyday context outside of unstructured play. Studying interactions in a naturalistic setting shows the parent that skills such as attunement and synchrony with her infant are applicable to every interaction between them.

The therapist makes effective use of 6 and 7 during the normal routine of a mealtime in order to demonstrate to the parent that skills such as attunement and synchrony (e.g., turn taking) with her infant are applicable to every interaction between them (i.e., not just during structured play). The therapist focuses on the positive interactions between parent and infant, with reference to eye contact, sharing of experiences and inferences of intentionality on the part of the parent. Methods, described 1 to 5 above, are also used effectively by the therapist.

Session 5 Sharing Feelings

Theoretical Basis: *Affect matching*

Session 5 introduces a technique to enhance maternal empathy: inviting the parent to speak as if she herself were the infant. This is carried out using a video clip of face-to-face interaction to encourage affect matching.

The therapist encourages the parent to speak as if she herself was the infant (8 Baby Talk) in order to enhance maternal empathy and encourage affect matching. The therapist models this narration technique for two minutes by providing infant's thoughts, emotions and behaviours through interpreting the infant's facial expressions and non-verbal cues. The parent is then encouraged to take over for two minutes and any discrepancies are discussed. The therapist also identifies at least two Sensitivity Chains (6), applies a Corrective Message (7) and continues effect use of methods 1 to 5 described above.

Session 6 Sharing Talk

Theoretical Focus: Communication

Reciprocal vocalisations in a social context require a contingent, attuned response from the parent. This session builds on previous sessions by engaging the mother in the more subtle aspects of vocal and non-vocal communication as viewed from the context of a more structured triadic-play interaction involving book reading.

The therapist emphasises how the parent and infant communicate with each other by describing their vocal communication (talk, social babble, and turn-taking) and non-verbal behaviours, including eye contact. By watching the "Funny Sound Game" the therapist directs the parent's attention to how the infant responded to being imitated, and also discuss the parent's own experience during the activity. The therapist also identifies at least two Sensitivity Chains (6), applies a Corrective Message (7) and continues effect use of methods 1 to 5 described above.

I-BASIS Analysis Plan – 16TH April 2014

Brief description of trial objectives

Primary: To test the impact of a parent-mediated intervention for infants at high risk of autism using an experimental trial. This will be a pilot study testing a theory and evidence based intervention for subject acceptability, adherence and initial evidence of effect within a UK context.

Secondary: To use this intervention study to test hypotheses about the sensitivity to environmental change of selected potential risk markers for later autism namely, a) markers of behavioural atypicality, b) neurophysiological biomarkers.

Trial design

Design: Two group parallel arm comparing treatment with a therapist led parent-mediated behavioural intervention administered over 20 weeks with no treatment. Research staff responsible for all baseline and endpoint measurement were blind to treatment allocation. Baseline assessment: 9 months. Interim outcome assessment: 14 months. Follow-up assessments at 24 and 36 months.

Randomization: Individual randomisation 1:1 to Intervention or No intervention. One binary stratification factor Centre (London, Manchester). Permuted block approach within the 2 strata with random block sizes of 4 or 6. Target sample size was 50 (25 each arm); actual recruitment was 54 (28 intervention arm, 26 control arm).

Baseline comparability

The treatment and control groups will be compared on the following variables to check for successful randomisation balance.

Demographic variables

Age at baseline visit: in months and days. This is especially important for comparisons that require control for age.

Gender: male and female coded '0' and '1' respectively

Maternal Ethnicity: coded Caucasian '0' and other '1'

Household annual income: coded '0' <£40,000 and '1' £40,000+

Maternal education: coded '0' for below degree level, '1' for degree level or above

Maternal medical history: coded '0' for no disorder history, '1' for any known medical conditions

ASD/Typically developing siblings: coded '0' for at least one typically developing sibling and '1' for older sibling(s) with ASD only.

In addition, balance will be assessed for all baseline values of the primary and secondary outcome variables and baseline Mullen non-verbal T-score.

CONSORT Diagram

A CONSORT diagram will be presented showing consenting, exclusions, randomization, and features of drop-out, non-compliance and data completion.

Measures

Manchester Assessment of Caregiver-Infant interaction (MACI, Wan et al., 2012, 2013): baseline & outcome

Parent-infant free-play interaction was filmed at each visit and parents asked to play with their child as they would usually at home with toys (as provided) if they so wished. Six

minute clips were later coded using the MACI by a trained coder, blind to family information. The MACI has been modified from validated measures to suit our study group by combining the brevity/age appropriateness of the Global Rating Scales of Mother-Infant Interaction and the autism-specificity and format of the ‘Coding of Attachment-Related Parenting -for use with children with Autism’. The measure comprises 2 caregiver scales (sensitive responsiveness, non-directiveness), 3 infant scales (attentiveness to caregiver, positive affect, liveliness), and 2 dyadic items (synchrony/mutuality, quality of engagement). Each item involves rating on a 7-point scale. For the trial analysis *infant attentiveness* is the primary outcome, and *mutuality*, *parent non-directiveness*, *parent sensitive responsiveness* and *infant positive affect* are secondary outcomes.

Hypothesis: all measures will be *increased* by the intervention.

The Autism Observation Scale for Infants (AOSI, Bryson et al. 2008): baseline & outcome

The AOSI is a semi-structured observational assessment used to assess the early behavioural expression ASD. The AOSI examines precursors of later autism symptoms such as response to name, social reciprocity and imitation, as well as items assessing motor and sensory skills. Behaviours are coded are 0, 1, 2 (and in some cases 3) with a higher score indicating a greater level of autistic-like atypicality.

The *total score* is calculated by adding together scores from all items in the battery, excluding items 19, 20 and 21.

Hypothesis: the total score will be *decreased* by the intervention.

Mullen Scales of Early Learning (MSEL; Mullen, 1995): baseline & outcome

The *MSEL* is a standardised developmental assessment, which examines early motor and cognitive development from 0-68 months. The assessment is comprised of five subscales: gross motor (GM), visual reception (VR), fine motor (FM), receptive language (RL) and expressive language (EL). A composite non-verbal T-score will be calculated as (VR+FM)/2

Analysis will use *RL* and *EL* t-scores unless the data description phase gives evidence of floor-effects, when raw scores will be used together with covariation for age at assessment.

Hypothesis: both RL and EL will be *increased* by the intervention.

Vineland Adaptive Behavior Scales (VABS-II; Sparrow et al., 2005*): baseline & outcome*

The VABS-II is a parent report measure of daily living skills, which yields scores across a range of ages and competency levels for a range of subscales (e.g. motor, language, play, social skills). Items are scored on a three-point scale: Never ‘0’, Sometimes ‘1’ or Usually ‘2’, with Don’t Know or No Opportunity responses also possible. Don’t Know and No Opportunity are rescored as ‘1’, provided there are not more than two such responses within a subscale.

Analysis will use v-scores for *communication* and *socialization*, unless the data description phase gives evidence of floor effects, when raw scores will be used together with covariation for age at assessment.

Hypothesis: Both *communication* and *socialization* will be *increased* by the intervention.

MacArthur-Bates Communicative Development Inventory (MCDI; Fenson et al., 1993): baseline & outcome

The MCDI *words and gestures* is a standardised parent report measure of vocabulary. In the first half of the questionnaire, parents fill in the number of words understood, or understood and said for words in different semantic categories, i.e., action words, people etc., with a total of 396 words. The second half of the questionnaire asks about communicative and symbolic gestures that the child uses. For gesture subsections with responses ‘not yet’,

'sometimes' and 'often' a binary response of 0 for 'not yet' and 1 for 'sometimes' or 'often' is recorded. Other subsections with 'yes'/'no' responses are also coded '0' and '1'.

We will use the total of '*understands and says'* for expressive vocabulary and the *combined 'understands' with 'understands and says' score* for receptive vocabulary. A *total score* for *gestures* will also be calculated.

Hypothesis: Both *receptive* and *expressive vocabulary* and *gesture score* will be *increased* by the intervention.

ERP: Mismatch response: outcome

This ERP paradigm consists in presenting sounds in an oddball way: 77% of the stimuli are /u/ vowels (standards), and two different types of infrequent sounds are presented each with 11.5% probability. The deviants are /u/ vowels with a different pitch to the one of the standards (pitch deviants), and /i/ vowels with the same pitch as the standards (speech deviants). At least 2 standards succeeded each deviant. The inter-stimulus interval was fixed at 700 ms. Stimulus presentation was stopped after 700 stimuli were heard or when the infant fussed out. During stimulus presentation the infant was seated on caregiver's lap and an experimenter blew bubbles, to direct infant's attention away from the auditory stimulation (condition under which differences between ASD and TD were found, Lepistö et al, 2009).

Brain electrical activity was measured using an EGI 128- channel Hydrocel Sensor Net. The reference electrode at recording was the vertex (Cz in the conventional 10/20 system). The EEG was filtered online with 0.1–200-Hz bandpass, digitized at 500-Hz sampling rate. Continuous data were filtered offline with a 0.3-40 Hz band-pass filter. Epochs of 800-ms duration, including 100-ms prestimulus interval, were extracted for each stimulus. The first 2 epochs were excluded from the analysis and only standard trials preceding the deviant trials were analysed. This ensures that a comparable number of trials were included in the analysis of standard and deviant responses and also that local fluctuation of attention affect both types of stimuli similarly. Within each trial electrodes showing voltage fluctuations larger than 200microV were marked as bad. Trials with more than 20 bad channels were marked as bad. Visual inspection of this automated artifact rejection led to further channel and trial removal. Only participants with at least 15 trials in each condition were included in further analysis. Bad channels data was interpolated using spherical splines interpolation (Perrin et al, 1987). All trials within a condition and a participant were averaged. The averaged response was baseline corrected to the 100 ms pre-stimulus and re-referenced to an average reference. Deviant sounds evoke a more positive response than standard sounds over central, parietal and frontal electrodes, starting from around 120 ms. The first part of this response, over central and parietal electrodes is believed to reflect the dishabituation of auditory cortex response to the change in sound properties (Dehaene-Lambertz & Gliga, 2004). A frontal positivity 400-600 ms after the stimulus onset, similar to a p3 response measured in children 2 year old and older (Kushnerenko et al, 2002) reflects attention orienting. For the early time window (p1) voltage was averaged between 120 and 320 ms and over 17 central and parietal channels: 112, 106, 105, 111, 13, 7, 30, 29, 36, 104, 42, 37, 31, 80, 87, 93, 6. For the late time window (p3) voltage was averaged between 400 and 600 ms and over 18 frontal and central channels: 112, 106, 105, 111, 13, 7, 30, 29, 6, 20, 12, 5, 118, 11, 24, 36, 104, 124.

Responses within both earlier and later time windows have been found to differentiate between individuals with autism or at-risk for autism and controls (p3 and attention orienting Lepistö et al, 2005; Whitehouse & Bishop, 2008; p1/n1 and perceptual processing Guiraud et al, 2011; Kuhl et al, 2005; Lepisto et al, 2006). More consistent group differences are found when analysing response to speech sounds (Lepistö et al, 2005; reviewed in O'Connor, 2012). We therefore decided to only analyse the response to a change in vowel, within both the early and the late time window. To look at the response induced by the detection vowel change, a difference between the averaged values for the *speech deviant* and the *standard* will be

calculated. We expect this difference (for both the late time window and the early time window) to be stronger in the intervened group.

The *amplitude* of the *speech difference* wave form at ~*120ms* and *400-600ms*, covarying for age at assessment. We will test whether there were any differences in the number of trials contributed by the two groups.

Hypothesis: the positive amplitude of the speech difference at ~*120ms* and *400-600ms* will be *increased* by the intervention.

The gap-overlap task: baseline & outcome

Stimuli were presented on a Tobii 1750/TX120 eyetracker subtending 24˚; stimuli were presented gaze-contingently using Matlab and the Talk2Tobii toolbox. Stimuli were presented in five blocks interspersed with other elements of the testing battery. The first four blocks lasted 12 trials per block, with an 8-second video reward presented between trials 6 and 7; the fifth block continued until 12 usable trials per condition had been presented, until the infant became fussy or until 80 trials had elapsed. After fixating a Central Stimulus (a cartoon clock/balloon, subtending 4.5˚) a Lateral Target (a cartoon cloud, subtending 3˚) was presented to the left or right at an eccentricity of 6˚ following a delay of 150ms; when the participant looked to the LT they received a brief audio visual reward. Reaction times were assessed in three conditions: (1) Gap: LT presented 200ms after the offset of the CS; (2) Baseline: CT offset simultaneous with LT onset; (3) Overlap: CS remained on screen when LT was presented. The start of each trial and the reward was automatically triggered online when gaze landed in the relevant area of the screen, following custom routines implemented in Matlab/Psychtoolbox. Reaction time was calculated as the time elapsed between LT appearance and the reported position of gaze entering the LT position (a 9˚ box around the LT). Trials were excluded from analysis if there was a period of more than 60ms of continuous data loss between peripheral stimulus onset and the eyes entering the position of the lateral target; if the eyes were not fixating the central stimulus at the time of peripheral stimulus onset; if the child did not make a saccade to the lateral target within 2 seconds of peripheral stimulus onset; or if the child disengaged from the screen within 2 seconds of peripheral stimulus onset without first saccading to the peripheral stimulus.

Subsequently, mean reaction time per condition was calculated, excluding reaction times less than 100ms (thought to be less than the minimum latency required to program a saccade in response a stimulus appearing) and reaction times greater than 1200ms as they are thought not to represent exogenously driven reactions to the stimulus presentation (e.g. Elsabbagh et al., 2013).

For this analysis *disengagement* will be calculated for each infant as the mean reaction time for valid overlap trials minus mean reaction time for valid baseline trials. Number of valid trials in the two conditions will be reported to ensure this does not differ between groups.

Hypothesis: *reaction times for disengagement* will be *reduced* by the intervention.

Analysis overview

Treatment Blinding: The analysis plan has been written prior to linkage of treatment variable to endpoint data and treatment unblinding. All analyses will be undertaken blind to treatment group coding. Analyses will be undertaken in Stata (StataCorp. 2011) 12.0.

Data description: The patterns of availability of baseline and follow-up data will be summarised separately for the two treatment groups for each measure. Descriptive summary statistics of means and standard deviations, and proportions, will be presented. No statistical

significance tests or confidence interval will be calculated for the difference between randomised groups on any participant level baseline variables. The randomisation of intervention groups to participants should have ensured that any imbalance over all measured and unmeasured baseline characteristics is due to chance.
Missing data: The pattern of missing data, and the reasons for it, will described. Analyses of data involving missing outcome measures will be undertaken wherever possible by full information maximum likelihood. The primary approach to missing baseline data will be approached using multiple imputation, carried out using iterative chained equation approach as implemented in Stata. Both ML and MI allow for potentially selective attrition. Where, for any analysis, complete data cases falls below 70%, complete-case only analyses will also be reported.
Treatment Compliance/Adherence: The mean and range of the proportion of treatment sessions attended will be reported and an adherence score derived from therapist sessional records of engagement and understanding. The criterion for minimum adherence is 6 sessions. We pre-specify criteria for high versus low adherence score groups to test the effect of good adherence on treatment effect.
Effect Estimators: Analysis of the intervention effect element of the study will initially use an intention to treat (ITT) principle i.e. participants will be analysed in the groups to which they were randomised irrespective of treatment amount or treatment quality received, utilising all available follow-up data from all randomised participants. Analysis will compare endpoint group effects using apriori hypotheses related to each measurement domain. Where the mean treatment compliance falls below 80% the Local Average Treatment Effect (LATE) will also be reported for the primary outcome. LATE continues to exploit the randomized to treatment allocation undertaken, and are also valuable in obtaining estimates of mediation unbiased by residual confounding.
Control Variables: Analyses will control for variables for which baseline imbalance is evident. Those variables for which the likely linearity of effects would be questionable, e.g. where baseline measures show evidence of floor effects, no control will be attempted.
Significance and Precision: The analyses specified will use a 5% (2-sided) significance level with 95% confidence intervals. Given the modest sample size and concern about the appropriateness of asymptotic estimators of precision, confidence intervals will be obtained by bootstrap resampling. To reduce multiple testing, significance tests for measures with multiple sub-scores will use global tests with multiple degrees of freedom.
Model checking: For models assuming continuous responses Q-Q plots of residuals will be examined.

Primary analysis
The primary outcome measure is infant attentiveness from the MACI. This is a revision to that originally proposed. The choice in primary outcome (MACI 'infant attentiveness to caregiver') is a change from the original proposal to look at the AOSI. The change in nominated primary outcome was made on the basis of results from our Preschool Autism Communication Trial (Green et al 2010; Pickles et al 2014 in press), which have emerged since the original iBASIS analysis plan was written in 2009. This trial, of a similar intervention to iBASIS but in children diagnosed with autism in the preschool years, showed a main effect on *child communication initiations* to the parent. Change in these communication initiations mediated change in child *autism behaviours,* but this latter was much attenuated and did not reach significance. Since these causal effects are consistent with developmental theory we consider now that the most appropriate choice of primary outcome for this prodromal intervention is the measure of *infant attentiveness to parent*. In our prospective developmental studies, quality of infant attentiveness at 12 months predicts later autism emergence at 3yrs. The AOSI measure (analogous to the autism behaviours at the later age) now becomes a secondary outcome. Both measures are researcher-rated, blind to treatment allocation.

Dependent variable: Regression of 14-month infant attentiveness on:
Independent variables: Treatment (Intervention or Control); Age at testing; Baseline (9 month) infant attentiveness and any other baseline variables for which imbalance has been found.

Secondary analysis

1) AOSI: Total score: The AOSI total score is a continuous variable and will be analysed using regression, with intervention group (treatment vs. control) as the predictor and covarying for baseline total score and age at testing.

2) MSEL: Receptive and Expressive raw scores: The distribution so of the endpoint MSEL RL and EL t- and raw scores will be examined for evidence of floor effects. In their absence the selected response (t-score or raw score) will be analysed as a two-outcome regression using an estimating equations approach with unstructured working correlation matrix (equivalent to MANOVA in complete data case) with a 2df significance test reported. Where floor effects are evident even for raw scores, separate tobit regressions will be estimated. Analyses will covary for age and imbalanced baseline variables, but where floor effects are evident, will not covary for baseline language scores.

3) MCDI: Receptive (understands + understands and says) and Expressive (understands and says) vocabulary; Total gesture score. For receptive and expressive vocabulary, analysis will follow the same pattern as for MSEL. However, there will be no covariation for baseline scores. Total gesture score will be analysed as a continuous variable using ANCOVA, with intervention group (treatment vs. control) as the predictor and covarying for baseline total score and age.

4) VABS: Communication and Socialization standardized subscale scores: The distribution of the endpoint VABS communication and socialization v- and raw scores will be examined for evidence of floor effects. In their absence the selected response (standard V-score or raw score) will be analysed as a two-outcome regression using an estimating equations approach with unstructured working correlation matrix (equivalent to MANOVA in complete data case) with a 2df significance test reported. Where floor effects are evident even for raw scores, separate tobit regressions will be estimated. Analyses will covary for age and imbalanced baseline variables, but where floor effects are evident, will not covary for baseline language score.

5) MACI: Parent sensitive responsiveness, parent non-directiveness, infant positive affect, mutuality. These will be analyzed as a four-outcome regression using an estimating equations approach with unstructured working correlation matrix (equivalent to MANOVA in complete data case) with a 4df significance test reported, covarying for baseline measures and imbalanced baseline variables.

6) GAP task: disengagement reaction time. Disengagement (reaction time/ms) will be analysed using regression covarying for baseline disengagement score and age at testing.

7) ERP MMN. Data from the *speech difference* wave form at *~120ms* and *400-600ms* will be analysed as a two-outcome regression using an estimating equations approach with unstructured working correlation matrix with a 2df significance test reported. Analyses will covary for age and imbalanced baseline variables.

Data analysis plan: 24 months

Primary analysis

The primary outcome measure is infant attentiveness to parent from MACI.

Dependent variable: Regression of 24 month CIA covarying for baseline infant attentiveness score.

Independent variables: Treatment (Intervention or Control); age at assessment and any imbalanced baseline variables.

Secondary analysis

1) ADOS Severity score (Gotham, Lord & Pickles, 2009)

2) Q-Chat: Total score

3) MSEL: Receptive and Expressive raw scores

4) MCDI: Receptive (understands + understands and says) and Expressive (understands and says) vocabulary

5) VABS: Communication and Socialization standardized subscale scores

6) DCMA: Proportion of parental acts that were synchronous.

7) MACI subscales as per 14 month analysis i.e. infant attentiveness; parent sensitive responsiveness, parent non-directiveness, infant positive affect, mutuality; parent-child joint attention

Analyses will follow the pattern specified for analogous 14-month measures.

Data analysis plan: 36 months

Primary outcome and analysis

The primary outcome measure is the ADOS severity score, analysed by regression covarying for baseline AOSI score and variables in baseline imbalance.

Secondary Outcomes: to be specified.

Analyses will follow the pattern specified for analogous 14-month measures.

References

Wan, M. W., Green, J., Elsabbagh, M., Johnson, M., Charman, T., Plummer, F. and The BASIS Team. (2012). Parent-infant interaction in infant siblings at risk of autism. *Research in Developmental Disabilities, 33,* 924-932.

Wan, M. W., Green, J., Elsabbagh, M., Johnson, M. H., Plummer, F., Charman, T., & The BASIS Team. (2013). Quality of interaction between at-risk infants and caregiver at 12-15 months is associated with three year autism outcome. *Journal of Child Psychology and Psychiatry, 54,* 763-771.

Mullen, E. M. (1995). *Mullen Scales of Early Learning* (AGS ed.). Circle Pines, MN: American Guidance Service Inc.

Sparrow, S.S., Cicchetti, D.V., & Balla, D.A. (2005). *Vineland Adaptive Behavior Scales. 2nd ed*. Oxford: NCS Pearson, Inc.

Bryson, S., Zwaigenbaum, L., McDermott, C., Rombough, V. and Brian, J. (2008). The Autism Observation Scale for Infants: scale development and reliability data. *JADD, 38,* 731-738.

Fenson, L., Dale, P. S., Reznick, J. S., Thal, D., Bates, E., Hartung, J. P. et al. (1993). *MacArthur Communicative Development Inventories*. Baltimore: Paul H. Brokes Publishing Co.

Lepistö, T., Kuitunen, A., Sussman, E., Saalasti, S., Jansson-Verkasalo, E., Nieminen-von Wendt, T., & Kujala, T. (2009). Auditory stream segregation in children with Asperger syndrome. *Biological psychology*, *82*(3), 301-307.

Perrin, F., Pernier, J., Bertnard, O., Giard, M. H., & Echallier, J. F. (1987). Mapping of scalp potentials by surface spline interpolation.*Electroencephalography and clinical neurophysiology*, *66*(1), 75-81.

Dehaene-Lambertz, G., & Gliga, T. (2004). Common neural basis for phoneme processing in infants and adults. *Journal of Cognitive Neuroscience*, *16*(8), 1375-1387.

Kushnerenko, E., Ceponiene, R., Balan, P., Fellman, V., & Näätänen, R. (2002). Maturation of the auditory change detection response in infants: a longitudinal ERP study. *Neuroreport*, *13*(15), 1843-1848.

Lepistö, T., Kujala, T., Vanhala, R., Alku, P., Huotilainen, M., & Näätänen, R. (2005). The discrimination of and orienting to speech and non-speech sounds in children with autism. *Brain research*, *1066*(1), 147-157.

Whitehouse, A. J., & Bishop, D. V. (2008). Do children with autism ‘switch off’ to speech sounds? An investigation using event- related potentials. *Developmental Science*, *11*(4), 516-524.

Guiraud, J. A., Kushnerenko, E., Tomalski, P., Davies, K., Ribeiro, H., Johnson, M. H., & BASIS Team. (2011). Differential habituation to repeated sounds in infants at high risk for autism. *Neuroreport*, *22*(16), 845-849.

Kuhl, P. K., Coffey- Corina, S., Padden, D., & Dawson, G. (2005). Links between social and linguistic processing of speech in preschool children with autism: behavioral and electrophysiological measures. *Developmental science*,*8*(1), F1-F12.

Lepistö, T., Silokallio, S., Nieminen-von Wendt, T., Alku, P., Näätänen, R., & Kujala, T. (2006). Auditory perception and attention as reflected by the brain event-related potentials in children with Asperger syndrome. *Clinical Neurophysiology*, *117*(10), 2161-2171.

O’Connor, K. (2012). Auditory processing in autism spectrum disorder: a review. *Neuroscience & Biobehavioral Reviews*, *36*(2), 836-854.

Elsabbagh, M., Fernandes, J., Webb, S. J., Dawson, G., Charman, T., Johnson, M. H., and The BASIS Team. (2013). Disengagement of visual attention in infancy is associated with emerging autism in toddlerhood. *Biological Psychiatry, 74,* 189-194.

Green, J., Charman, T., McConachie, H., Aldred, C., Slonims, V., Howlin, P. et al. (2010). Parent-mediated communication-focused treatment in children with autism (PACT): A randomised controlled trial. *Lancet, 375,* 2152–2160.

Gotham, K., Pickles, A. and Lord, C. (2009). Standardizing ADOS scores for a measure of severity in autism spectrum disorders. *JADD, 39,* 693-705.
